# Nosocomial surveillance of multidrug-resistant *Acinetobacter baumannii*: a genomic epidemiological study

**DOI:** 10.1128/spectrum.02207-23

**Published:** 2024-01-10

**Authors:** Zhimei Duan, Xuming Li, Song Li, Hui Zhou, Long Hu, Han Xia, Lixin Xie, Fei Xie

**Affiliations:** 1 College of Pulmonary and Critical Care Medicine, Chinese PLA General Hospital, Beijing, China; 2 Department of Scientific Affairs, Hugobiotech Co., Ltd., Beijing, China; Post Graduate Institute of Medical Education and Research, Chandigarh, India

**Keywords:** *Acinetobacter baumannii*, whole genome sequencing, antimicrobial resistance testing, genome typing, epidemiology, population genomics

## Abstract

*Acinetobacter baumannii* is a major opportunistic pathogen causing hospital-acquired infections, and it is imperative to comprehend its evolutionary and epidemiological dynamics in hospitals to prevent and control nosocomial transmission. Here, we present a comprehensive genomic epidemiological study involving the genomic sequencing and antibiotic resistance profiling of 634 *A*. *baumannii* strains isolated from seven intensive care units (ICUs) of a Chinese general hospital over 2 consecutive years. Our study reveals that ST2 is highly dominant (90.54%) in the ICUs, with 98.90% of the ST2 exhibiting multidrug resistant or extensively drug resistant. Phylogenetic analyses of newly sequenced genomes and public data suggest that nosocomial isolates originated outside the hospital but evolved inside. The major lineages appear to be stable, with 9 of the 28 identified nosocomial epidemic clones infecting over 60% of the affected patients. However, outbreaks of two highly evolved clones have been observed in different hospitals, suggesting significant inter-hospital transmission chains. By coupling patient medical records and genomic divergence of the ST2, we found that cross-ward patient transfer played a crucial role in pathogen’s nosocomial transmission. Additionally, we identified 831 potential adaptive evolutionary loci and 44 associated genes by grouping and comparing the genomes of clones with different prevalence. Overall, our study provides a comprehensive and contemporary survey on the epidemiology and genomic evolution of *A. baumannii* in a large Chinese general hospital. These findings shed light on the nosocomial evolution and transmission of *A. baumannii* and offers valuable information for transmission prevention and antibiotic therapy.

**IMPORTANCE:**

This study delved into the genomic evolution and transmission of nosocomial *Acinetobacter baumannii* on a large scale, spanning both an extended time period and the largest sample size to date. Through molecular epidemiological investigations based on genomics, we can directly trace the origin of the pathogen, detecting and monitoring outbreaks of infectious diseases in a timely manner, and ensuring public health safety. In addition, this study also collects a large amount of genomic and antibiotic resistance detection data, which is helpful for phenotype prediction based on genomic sequencing. It enables patients to receive personalized antibiotic treatment quickly, helps doctors select antibiotics more accurately, and contributes to reducing the use of antibiotics and lowering the risk of antibiotic resistance development.

## INTRODUCTION

*Acinetobacter baumannii*, a Gram-negative bacterium that commonly exists in natural environments, has evolved to be a worldwide prevalent pathogen that causes critical nosocomial infections in recent decades. The emergence and expansion of multidrug-resistant *A. baumannii* (MDRAB), particularly carbapenem-resistant *A. baumannii* (CRAB), has greatly intensified the difficulties in clinical *A. baumannii* infection (ABI) prevention and control. A meta-analysis has estimated the global prevalence of MDRAB in patients of *A. baumannii* causing hospital-acquired pneumonia or ventilator-associated pneumonia to be 79.9%, with associated mortality rates ranging from 37.2% to 48.1% (1). The China Antimicrobial Surveillance Network reported a substantial increase in the proportion of CRAB from 2005 to 2023, with incidence of imipenem resistance increasing from 31.0% to 78.6% and meropenem resistance from 39.0% to 79.5% (http://www.chinets.com/Data/GermYear).

In the early 1970s, the vast majority of *A. baumannii* isolates were sensitive to commonly used antibiotics (2). However, the widespread use of various antibiotics and genetic evolution, such as the acquisition of antibiotic resistance genes (ARGs) by horizontal gene transfer, have made *A. baumannii* resistant to a wide range of antibiotics (3, 4). Additionally, *A. baumannii* has adapted to the inhospitable hospital environments, such as prolonged periods of desiccation, routine disinfection regimes, and oxidative stress (5). Long hospital stays, catheter usage, and mechanical ventilation were previously thought to be related to MDRAB transmission, with immunocompromised and critically ill hosts predisposed to invasive infections (6, 7). As a result, MDRAB infections were more common in intensive care units (ICUs). Patients with ABI or colonization, healthcare workers, and environmental reservoirs are the main dissemination source in ICU (8), and the patient-to-patient transmission rate in the ICU was reported to be higher than 17% (9).

Multi-locus sequence typing (MLST) has been widely used to study the epidemiology of *A. baumannii*, and globally, ST1, ST2, ST25, and ST79 are the most common STs (10). ST2, which is pandemic in China and many other countries (10–12), is frequently associated with multidrug resistance and hyper-virulence (7, 12, 13). However, MLST is not suitable for studying strains with close genetic relationships, as it uses very limited genetic information. On the other hand, core genome-based typing provides significantly higher resolution and better understanding of the spread of closely related pathogens (14, 15). Meanwhile, the rapid adaptation of *A. baumannii* to changing environments and selection pressures depends on its ability to undergo rapid genomic changes. Comparative analyses based on the whole genome can provide detailed insights into genomic variations, including single nucleotide variations (SNVs) and variations in gene presence or absence, which can be instrumental in monitoring genomic features during adaptive evolution. However, large-scale genomic studies focusing on the nosocomial spread and evolution of *A. baumannii* are rare to date.

In this study, we conducted a genomic and epidemiological investigation of clinical *A. baumannii* isolates from multiple specimens of 487 hospitalized patients across seven ICUs, which covered a period of 2 years. The primary objective of our research was to explore the epidemiology of *A. baumannii* transmission within hospitals and monitoring the nosocomial evolutionary dynamics of the *A. baumannii* genome, aiming to gain a better understanding of the genetic basis of *A. baumannii*’s adaptation within the hospital setting and provide insights into the control of hospital-acquired transmissions.

## MATERIALS AND METHODS

### Strain collection and species identification

All of the preliminary identified *A. baumannii* isolates from seven different ICUs at the Chinese PLA General Hospital during January 2018 and December 2019 were included in this study (Table S1). Briefly, clinical specimens were inoculated on Columbia Blood Agar Plates (Autobio, China) and cultured at 37°C for 24 hours. Species identification of the isolated single colonies was performed based on VITEK-MS automatic microbiological analyzer (bioMérieux, France). To filter the contaminated samples, the isolates were screened a second time by species identification using VITEK-MS system after activation culture on China Blue Lactose Acid Agar Plates (Thermofisher Scientific, USA) before sequencing. A total of 724 clinical isolates were recovered for whole genome sequencing. The overall study designs and analysis work flow were plotted in Fig. S1.

### Antimicrobial susceptibility testing (AST)

AST was conducted using VITEK 2 Compact system. Nineteen antibiotics of 10 drug classes, including levofloxacin, ciprofloxacin, imipenem, cefepime, ceftriaxone, ceftazidime, cefuroxime axetil, cefuroxime, cefazolin, cefotetan, ampicillin-sulbactam, cefoperazone-sulbactam, ampicillin, aztreonam, gentamicin, tobramycin, trimethoprim-sulfamethoxazole, nitrofurantoin, and tigecycline, were used for AST analysis. MICs were interpreted following the Clinical & Laboratory Standards Institude (CLSI) M100 30th Edition. The isolates were classified into extensively drug resistant (XDR) or MDR referring to A.P. Magiorakos et al’s standard (16) (Table S2a through c).

### DNA extraction and genome sequencing

The DNA of 724 isolates were extracted by QIAamp DNA Mini Kit (Qiagen, Germany) and quantified by Qubit 2.0 Fluorometer (Thermo Fisher Scientific, USA) according to the standard operation manual. The sequencing libraries with insert size of 350 base pairs were constructed and sequenced on Illumina NovaSeq 6000 system (Illumina Inc., USA). Adapters were removed and reads with low quality or high percent of N were filtered.

### Genome assembly, annotation, serotype, and MLST typing

Firstly, species identification was performed on the clean data using Kraken2 (17) and 634 isolates were identified to be *A. baumannii*. Next, the clean reads of the 634 isolates were assembled by Unicycler (v0.4.8) (18) and the genes were predicted by Prokka (v1.14.6) (19) with the default parameters. The draft genomes were evaluated by BUSCO (v4.1.4) (20). The capsular polysaccharide (KL) and outer core of lipooligosaccharide (OCL) locus types were identified employing Kaptive2.0 (21). MLST typing was performed using MLST (v2.19.0) (https://github.com/tseemann/mlst) referring to PubMLST database (Pasteur scheme) (22) (Table S3). A total of 5,390 public *A. baumannii* genomes were downloaded from the National Center for Biotechnology Information (NCBI) genome database (https://www.ncbi.nlm.nih.gov) for phylogenetic analysis on December 2021, and the detailed information of these genomes was described in Table S4.

### Variants calling and phylogenetic analysis

The whole genome-based single nucleotide polymorphisms (SNPs) were called according to bwa (0.7.17)/GATK (v4.1.9.0) pipeline. The clean reads were mapped to reference genome using Burrows-Wheeler-Alignment (BWA) mem algorithm (23), referring to the complete genome of str. K09-14 (GCF_008632635.1), a clinical strain isolated from Malaysia. The variations were called by GATK (24) with parameters of “QD <2.0, MQ <40.0, FS >60.0, SOR >3.0, MQRankSum <−12.5, ReadPosRankSum <−8.0, DP <10” and were annotated by snpEff (v5.1) (25). SNVs with minimum allelic frequency (MAF) of 0.01 and minimum integrity of 0.9 were analyzed in the following study. A total of 103,389 high-quality SNPs were identified and 94,428 (91.32%) SNPs located in genic regions. Among them, 76.70% and 14.46% of the SNPs were annotated as synonymous variant and missense variant, respectively (Table S4a and b). 57.65% of SNPs were not rare mutations, with an MAF higher than 5%. The core genome was constructed by Parsnp (v.1.5.6) (26) with default parameters. Fifty-six thousand seventy-six (54.23%) SNPs were identified as being located in the core genome region (cSNP). The gene sets of 634 *A*. *baumannii* isolates were clustered by Roary (v.3.11.2) (27) with parameters of “-i 95 c mcl.” The phylogenetic tree of 634 private (Table S3) and 5,390 public (Table S4) genomes were constructed using the core genes identified by PhyloPhlAn (v3.0.60) (28). The maximum likelihood (ML) trees were constructed by RAxML (29) (https://raxml-ng.vital-it.ch/#/) with bootstrap of 1,000, and the trees were adjusted and plotted by iTOL (30) (https://itol.embl.de/).

### Association analysis between antibiotic resistance and SNP variations

Genome-wide association analysis was conducted to investigate the relationship between antibiotic resistance phenotypes and SNP genotypes within the ST2 using a linear mixed model implemented in GEMMA (version 0.98.3) (31). Sites associated with known antibiotic resistance phenotypes having a MAF of <0.01 were excluded from the analysis. The final SNP amounts of each sample used for analysis ranged from 7,521 to 7,717. For each antibiotic, both the transformed MICs and the specific MIC values were used as the phenotypes. The terms “Resistant,” “Intermediate,” and “Sensitive” were transformed to numerical values “2,” “1,” and “0,” respectively, for input. Results were reported as significant if they met the calculated permutation threshold of *P* < 0.001 and were consistent in both sets of results.

### Virulence factor genes (VFGs), ARGs, and epitopes identification

VFGs of the reference genome were annotated by the virulence factor database (VFDB) using online VFanalyzer (32), and VFGs of the other isolates were predicted using local BLASTp with parameters of “-qcov_hsp_perc 80.” ARGs were identified by using CARD (v3.1.4) (33) database with BLASTp parameters of “-qcov_hsp_perc 80.” Hits with an identity less than 80 were filtered in the VFGs and ARGs predictions. Epitopes are short peptides that play a crucial role in the immune response by being recognized by T-cell receptors or B-cell receptors. The epitopes were obtained from the Immune Epitope Database (https://www.iedb.org) with release version of v2-26. A total of 158 variant loci were identified in 177 epitopes across all isolates.

### Transmission history simulation

SCOTTI (34) was used to simulate the transmission events between patients. The sampling time, hospitalization wards, and admission-discharge dates of the hosts were reviewed for simulations. The pairwise cSNPs identified by Parsnp (v.1.5.6) (26) were used to conduct the simulation analysis. In this progress, “SCOTTI_generate_xml.py” was used to generate the input XML configuration files for BEAST2 MCC.tree analysis. Then, the trees were used for transmission network analysis using “Make_transmission_tree_alternative.py” and probabilities of transmissions between every pair of patients were calculated. Isolates from patients hospitalized in the same ICU were separately used to analyze the inter-ICU transmission. Isolates of same ST2 clades, which were considered as clones, were separately used to simulate the intra-ICU transmission. Only results following the below conditions were considered to be reliable: infection possibility of patients from the same ward appeared high in both inter-ward and ST2-clade simulations; or infection possibility of patients from same ward was low, but high within other ST2-clade groups. After simulating the transmission of isolates in both inter-ward and intra-ward dimensions, a total of 65 reliable links were obtained.

### ST2 isolates grouping

Nine clades spanning over 12 months were isolated and appeared in the last half of the sampling period, indicating a long continuity of propagation in patients (referred to as the Long Period Group, LPG). Meanwhile, seven clades were found to be temporarily prevalent, with a maximum time span of less than 8 months, and disappeared in the last half of the sampling period (referred to as the Short Period Group, SPG). An additional seven clades were defined as recent groups (RG) with a maximum time span of less than 12 months and appeared in the last half of the sampling period (Table S5).

### Statistical analysis

The statistical analyses were achieved using Rscript (v3.2.2). The difference of ST distributions within each ICU was tested using χ^2^ test, and *P* value <0.05 was considered statistically significant. The GO and KEGG enrichment analyses were tested by hypergeometric test.

## RESULTS

### Summary of isolates

**Fig 1:**
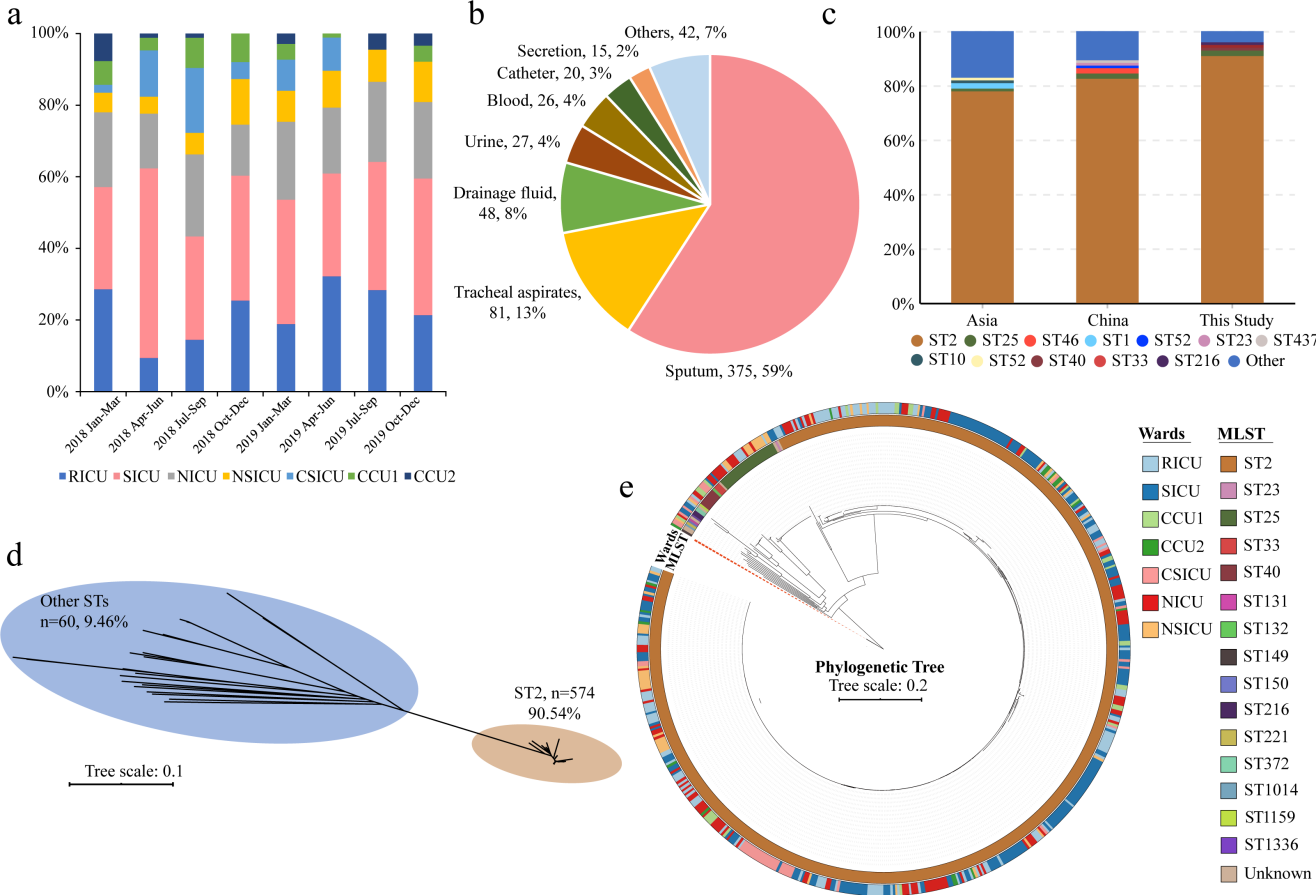
Phylogenetic relationships of the isolates. (a) Histogram showing the sampling time and sampled isolates ratio of each ward; the seven wards located in different floors of two buildings, including the respiratory intensive care unit (RICU) (26.49%), surgical intensive care unit (SICU) (26.28%), neurology intensive care unit (NICU) (22.79%), neurosurgery intensive care unit (NSICU) (9.45%), cardiosurgery intensive care unit (CSICU) (6.47%), Cardiovascular Care Unit 1 (CCU1) (5.75%) and cardiovascular care unit 2 (CCU2) (3.49%); (b) Pie graph shows the original source of isolated strains; (c) Comparison of ST components of the isolates within this study, China and Asia; (d) An unrooted ML tree showing the relationships of all isolates; all the ST2 isolates gather together and have a far genetic distance with the other STs; (e) A rooted ML phylogenetic tree showing the relationships of the isolates in this study; the red dash line shows the reference genome; the sequence types and origin of the isolates were differently colored.

**Fig 2:**
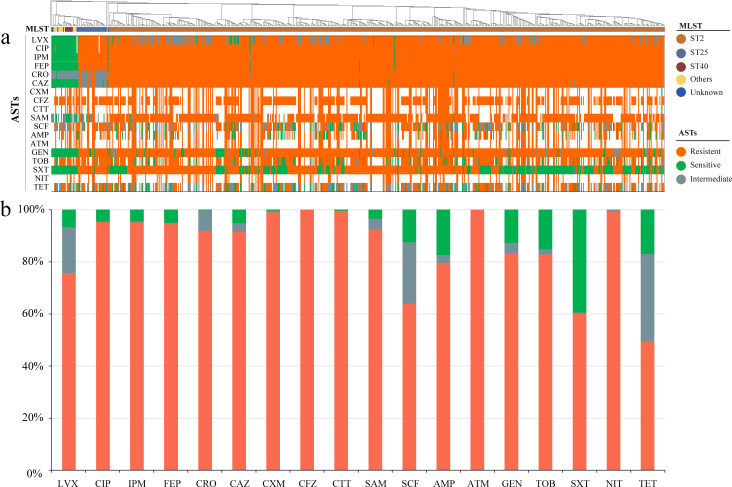
Antibiotic resistance of the *A. baumannii* isolates. (a) Phylogenetic distance of 634 isolates and the antibiotic resistance of each isolate. The sequence types of MLST were illustrated on the top and the rectangle tree shows phylogenetic relationship of each isolate; orange color, green color, and gray color in the ASTs region means “resistant,” “sensitive,” and “intermediate” to the corresponding antibiotics; the white blank of the ASTs region means the phenotype data were missing; the presence and absence of ARGs and VFGs were illustrated by different colors. LVX, levofloxacin; CIP, ciprofloxacin; IMP, imipenem; FEP, cefepime; CRO, ceftriaxone; CAZ, ceftazidime; CXM, cefuroxime; CFZ, cefazolin; CTT, cefotetan; SAM, ampicillin-sulbactam; SCF, cefoperazone-sulbactam; AMP, ampicillin; ATM, aztreonam; GEN, gentamicin; TOB, tobramycin; SXT, trimethoprim-sulfamethoxazole; NIT, nitrofurantoin; TET, tigecycline. (b) Statistics of the antibiotic resistance rate of each antibiotic of whole isolates.

The strains were isolated from various specimens, including sputum (59.15%), tracheal aspirates (12.78%), drainage fluid (7.57%), and others. Over 54.21% of the hosts were long-term hospitalized and aged patients (Fig. 1; Table 1; Table S1). Out of the 634 isolates, more than 380 (59.94%) were MDR, and 194 (30.60%) were XDR. The highest prevalence of non-susceptibility was to cephems (100%), followed by β-lactam combination agents (96.50%), carbapenems (95.43%), fluoroquinolones (95.41%), aminoglycosides (94.95%), folate pathway antagonists (60.57%), tetracyclines (58.36%), penams (42.59%), monobactams (32.81%), and nitrofuran (22.40%) (Fig. 2; Table S2a through c). The isolates mainly consist of three sequence types, including ST2 (90.54%), ST25 (4.57%), and ST40 (1.26%). Over 98.90% of the ST2 were MDR or XDR (Fig. S2 and S3). The distribution of ST2 did not significantly differ between wards (Fig. S4) and the phylogenetic tree demonstrated that ST2 are genetically distinct from other sequence types (Fig. 1d). The close genetic relatedness and widespread dispersion of ST2 across all wards suggest significant nosocomial cross-transmissions (Fig. 1e).

**TABLE 1:** Demographic information and characteristics of the *Acinetobacter baumannii* isolates

Item	All	RICU	SICU	NICU	NSICU	CSICU	CCU1	CCU2
No. of patients	487 (100%)	129 (26.49%)	128 (26.28%)	111 (22.79%)	46 (9.45%)	31 (6.37%)	28 (5.75%)	17 (3.49%)
Gender								
Male	365 (74.95%)	101 (78.29%)	96 (75.00%)	83 (74.77%)	32 (69.57%)	21 (67.74%)	20 (71.43%)	15 (88.24%)
Female	122 (25.05%)	28 (21.71%)	32 (25.00%)	28 (25.23%)	14 (30.43%)	10 (32.26%)	8 (28.57%)	2 (11.76%)
Age								
<50 years	132 (27.10%)	17 (13.18%)	52 (40.63%)	39 (35.14%)	10 (21.74%)	12 (38.71%)	1 (3.57%)	2 (11.76%)
50–70 years	169 (34.70%)	34 (26.36%)	50 (39.06%)	37 (33.33%)	24 (52.17%)	11 (35.48%)	10 (35.71%)	4 (23.53%)
>70 years	186 (38.19%)	78 (60.47%)	26 (20.31%)	35 (31.53%)	12 (26.09%)	8 (25.81%)	17 (60.71%)	11 (64.71%)
Average (years)	60	70	53	56	58	53	74	74
In hospital								
<28 days	221 (45.38%)	71 (55.04%)	51 (39.84%)	47 (42.34%)	14 (30.43%)	9 (29.03%)	17 (60.71%)	12 (70.59%)
>=28 days	264 (54.21%)	58 (44.96%)	77 (60.16%)	64 (57.66%)	30 (65.22%)	22 (70.97%)	11 (39.29%)	5 (29.41%)
No. of isolates	634 (100%)	141 (22.24%)	224 (35.33%)	125 (19.72%)	53 (8.36%)	45 (7.1%)	29 (4.57%)	17 (2.68%)
Source								
Sputum	375 (59.15%)	77 (54.61%)	98 (43.75%)	108 (86.40%)	46 (86.79%)	3 (6.67%)	27 (93.10%)	16 (94.12%)
Tracheal aspirates	81 (12.78%)	43 (30.50%)	5 (2.23%)	0 (0.00%)	0 (0.00%)	32 (71.11%)	0 (0.00%)	1 (5.88%)
Drainage fluid	48 (7.57%)	0 (0.00%)	48 (21.43%)	0 (0.00%)	0 (0.00%)	0 (0.00%)	0 (0.00%)	0 (0.00%)
Urine	27 (4.26%)	3 (2.13%)	14 (6.25%)	7 (5.60%)	3 (5.66%)	0 (0.00%)	0 (0.00%)	0 (0.00%)
Blood	26 (4.10%)	1 (0.71%)	15 (6.70%)	2 (1.60%)	1 (1.89%)	6 (13.33%)	1 (3.45%)	0 (0.00%)
Catheter	20 (3.15%)	2 (1.42%)	11 (4.91%)	4 (3.20%)	0 (0.00%)	2 (4.44%)	1 (3.45%)	0 (0.00%)
Secretion	15 (2.37%)	2 (1.42%)	12 (5.36%)	1 (0.80%)	0 (0.00%)	0 (0.00%)	0 (0.00%)	0 (0.00%)
Peritoneal fluid	9 (1.42%)	0 (0.00%)	8 (3.57%)	0 (0.00%)	0 (0.00%)	1 (2.22%)	0 (0.00%)	0 (0.00%)
Others	33 (5.21%)	13 (9.22%)	13 (5.80%)	3 (2.40%)	3 (5.66%)	1 (2.22%)	0 (0.00%)	0 (0.00%)
Sequence type								
ST2	574 (90.54%)	133 (94.33%)	215 (95.98%)	109 (87.20%)	39 (73.58%)	37 (82.22%)	25 (86.21%)	16 (94.12%)
ST25	29 (4.57%)	6 (4.26%)	0 (0.00%)	11 (8.80%)	12 (22.64%)	0 (0.00%)	0 (0.00%)	0 (0.00%)
ST40	8 (1.26%)	1 (0.71%)	0 (0.00%)	2 (1.60%)	0 (0.00%)	3 (6.67%)	2 (6.90%)	0 (0.00%)
Others	23 (3.63%)	1 (0.71%	9 (4.02%)	3 (2.40%	2 (3.77%	5 (11.11%)	2 (6.90%)	1 (5.88%)

Each ST-type OCL subtype exhibits a high degree of conservatism, with a one-to-one correspondence between ST types and OCL subtypes (Table S6a through c). To illustrate, ST2 aligns exclusively with OCL1, ST25 is uniquely linked to OCL6, and ST40 shares its correspondence with OCL6 as well. In contrast, KL subtypes display a more pronounced diversity, wherein a single ST type may be associated with various KL subtypes. For instance, ST2 demonstrates associations with 22 distinct KL subtypes, primarily encompassing KL3 (*n* = 147, 25.61%), KL2 (*n* = 88, 15.33%), KL210 (*n* = 85, 14.81%), and KL160 (*n* = 56, 9.75%).

### Overview of whole genomic variations

The average genome size of the isolates is 3.97 Mb, with an average of 3,838 coding genes per genome (Table S3). Of the 103,389 high-quality SNVs (referring to K09-14), 16.07% (16,610) were identified within ST2, which occupied approximately 0.42% of the whole genome (Table S7a and b). Additionally, fewer cSNPs (4,374, 7.80%) were identified between ST2 in the core genome region. These SNP variants were located within the coding regions of 937 genes, and 373 genes were found to be severely altered with variant loci occupied more than 1% of the coding regions (Table S8). These genes were enriched in 20 Gene Ontologies (GO) and 10 KEGG pathways, including GO:0046677 that responds to antibiotics (*P* = 0.0031) (Fig. S5) and KO01504 (*P* = 0.0051) related to antimicrobial resistance (Table S9; Fig. S6).

### Profiling of VFGs and ARGs of ST2.

**Fig 3:**
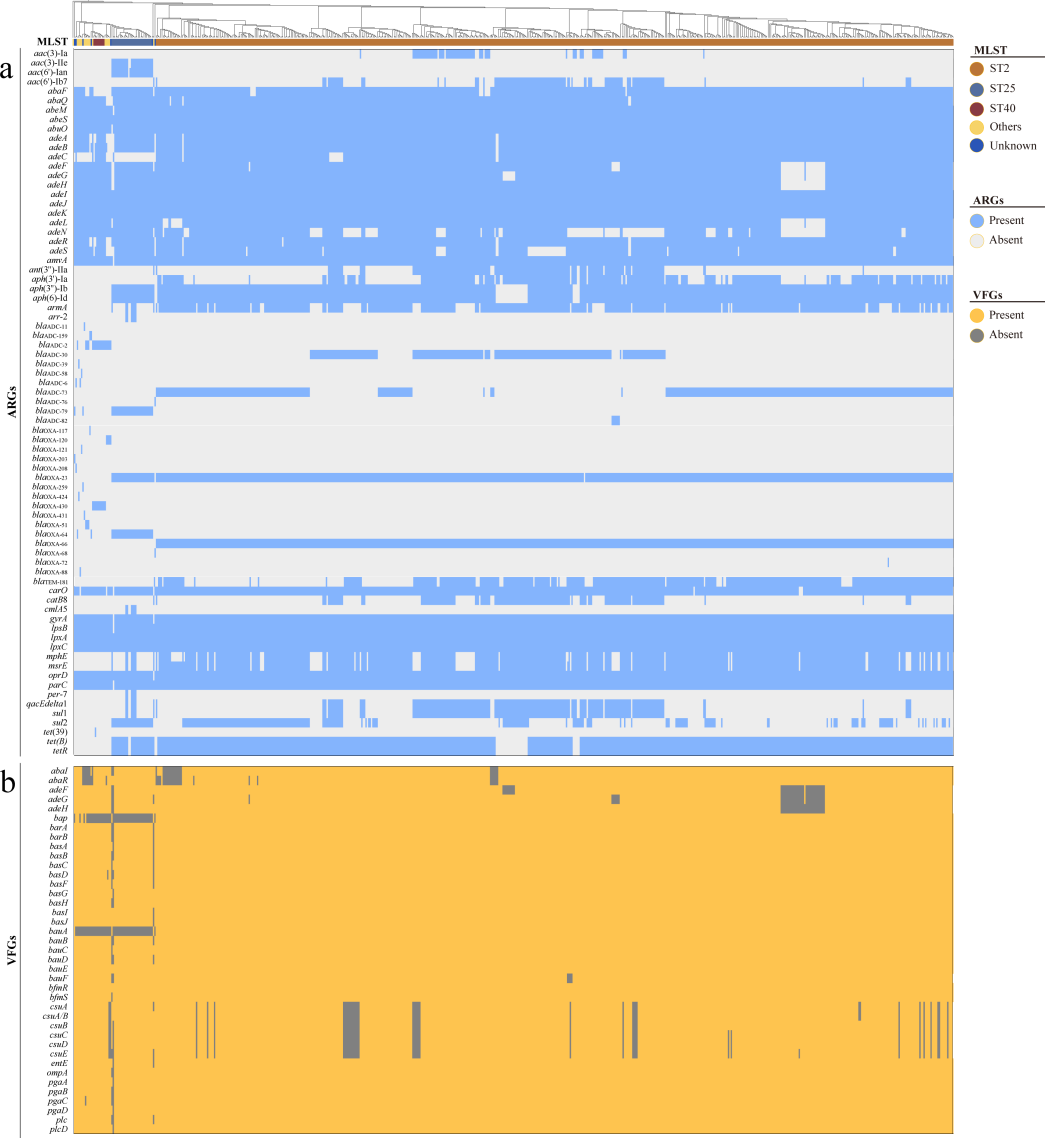
ARGs and VFGs profiling of the *A. baumannii* isolates. (a) ARGs profiling of 634 isolates; (b) VFGs profiling of 634 isolates. The sequence types of MLST were illustrated on the top and the rectangle tree shows phylogenetic relationship of each isolate.

The main beta-lactamase genes in ST2 are *bla*_OXA-23_ (99.65%), *bla*_OXA-66_ (99.82%), *bla*_TEM-181_ (67.19%), *adc*-73 (60.38%), and *adc*-30 (38.39%) (Fig. 3a; Table S10). The most abundant aminoglycoside antibiotic-related ARGs in ST2 are *aph*(3’’)-Ib (94.93%), *aph* (6)-Id (94.93%), *aph*(3’)-Ia (59.68%), *ant*(3’’)-IIa (32.98%), *aac*(6’)-Ib7 (22.33%), and *aac* (3)-Ia (12.04%). Over 99.82% of ST2 genomes contain resistance-nodulation-division (RND) type and major facilitator superfamily (MFS) type antibiotic efflux pump genes. Low frequencies of *sul*1 (34.38%) and *sul*2 (36.12%), which are related to sulfonamide or sulfone antibiotics, were observed. Other ARGs that are present in over 80% of the ST2 include *tet(B)* (94.93%), *tetR* (94.93%), *gyrA* (99.82%), *parC* (99.82%), *lpxA* (99.82%), *lpxC* (99.82%), *msrE* (87.95%), *mphE* (86.21%), *carO* (99.12%), and *oprD* (99.82%).

Overall, the mutation ratio was observed higher in coding regions than in non-coding regions (0.43% vs 0.31%, *P* < 0.001) across all ST2, suggesting that coding regions experienced stronger selective pressure (Table S11). Interestingly, the mutation loci ratio of ARGs was similar to that of coding regions as a whole (0.42% vs 0.42%, *P* = 0.486), but VFGs had a much higher mutation loci ratio than ARGs (2.00% vs 0.42%, *P* < 0.001). Conversely, ARGs (29/48, 60.42%) showed a higher rate of copy number variations (present over 99%) than VFGs (11/39, 28.20%) (Fig. 3; Fig. S7 and S8; Tables S10 and S12). Moreover, the copy numbers of ARGs and VFGs were mostly consistent within each clade. In addition, we identified 14 highly mutated VFGs and six highly mutated ARGs, with over 1% variant loci in the coding region (Table S13). 66.67% (4/6) of these ARGs, including *adeA*, *adeB*, *adeR,* and *adeS*, were relevant to glycylcycline and tetracycline antibiotic, and 64.29% (9/14) of the VFGs were correlated with immune evasion.

### Isolates originated outside the hospital and evolved inside

The MLST analysis of publicly available genomes showed that the *A. baumannii* ST types varied widely across continents and countries (Fig. S9; Table S14a). Remarkably, the proportion of ST2 in this study was much higher (90.54%) than the public data of China (86.0%), USA (67.0%), India (59.0%), Thailand (57.0%), Germany (46.0%), and other countries (Fig. 1c; Table S14b and c). The phylogenetic tree, comprising of 6,024 worldwide *A. baumannii* genomes, illustrated that the strains isolated from our hospital are universally separated by the public samples, suggesting that these private isolates have multiple origins (Fig. S10). Most of the private isolates are not uniformly distributed but clustered, suggesting that the majority of the clades are specific to the hospital. Furthermore, most of the neighboring strains were geographically isolated from Asia, indicating a regional dissemination of *A. baumannii* epidemiology.

**Fig 4:**
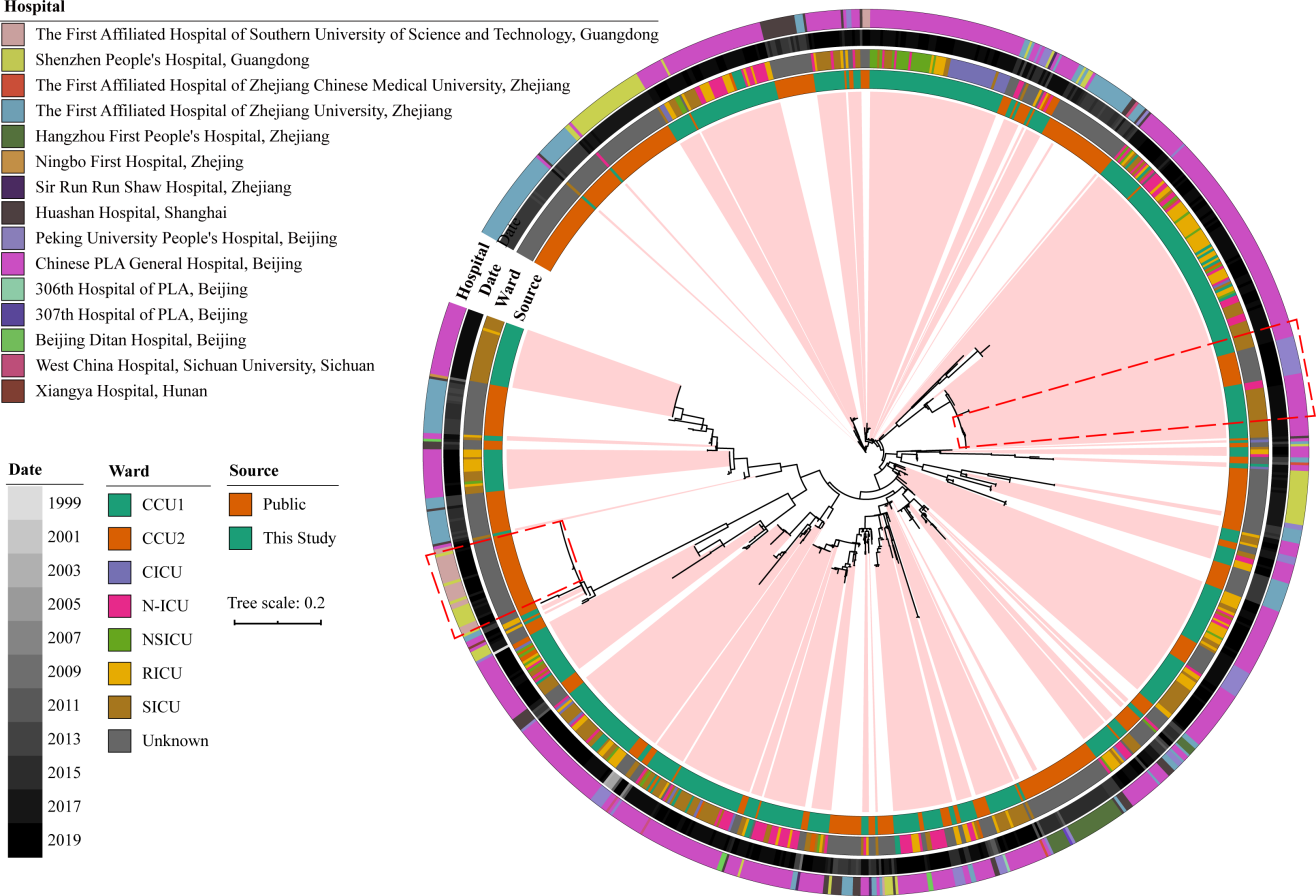
Phylogenetic tree showing the ST2 *A. baumannii* from hospitals in different provinces of China. The ML tree was constructed using protein sequence of the core genes of 6,024 *A*. *baumannii*; the outer circle shows the hospitals types, the release date, ward source, and the data source of the strains from outside to inside; isolated from Beijing was marked with light pink color; two highly evolved clones with co-appearance in different hospitals that located at same city were marked with red trapezoidal box.

Another phylogenetic tree of clinical ST2 from 15 hospitals located in six Chinese provinces revealed that closely related isolates from the same hospital tend to cluster together in the same clade (Fig. 4). Moreover, isolates from hospitals in the same province exhibit a trend of close genetic relatedness, implying a regional dissemination of the same clades. Thus, it can be inferred that the isolates originated outside the hospital and evolved inside, which is further supported by the phylogenetic tree of the entire set of available isolates from China (Fig. S11) and the ST2 isolates from China (Fig. S12).

Additionally, cross-transmission of the same clade among different hospitals was also observed. As shown in Fig. 3, the same clone strains have been identified at Chinese PLA General Hospital and Peking University People’s Hospital in Beijing, as well as at Shenzhen People’s Hospital and the First Affiliated Hospital of Southern University of Science and Technology in Guangdong, Shenzhen, which implies the existence of cross-hospital transmission events.

### Detailed typing and epidemic history of ST2 in hospital

**Fig 5:**
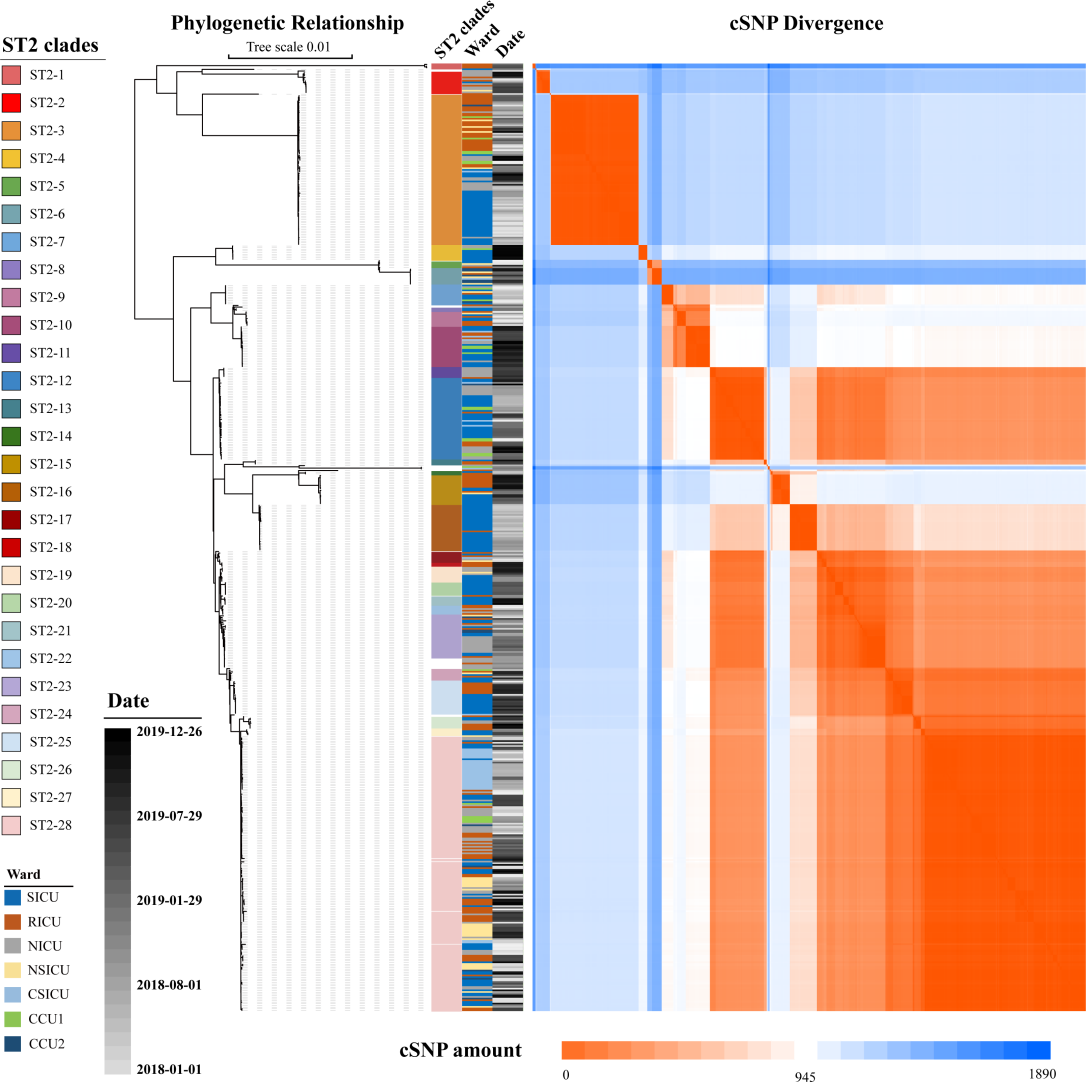
Genotyping of ST2 strains. The left phylogenetic tree shows the phylogenetic relationship of the ST2 strains; the middle columns show the information of isolates, including ST2 clades, ward, and isolated date; the right heatmap shows the cSNP divergence within each isolate.

After combining phylogenetic relationships with a cross matrix of the cSNP divergence of the entire ST2 in this study (Fig. 5; Table S15), a total of 28 clonal clades were clustered, which included 94.66% (550/574) isolates from 411 (84.39%) patients, with a genomic distance cutoff of 20 cSNPs (Table S5) (35). In total, 4,374 cSNPs were identified between the ST2, and within the 28 clades, 1,343 cSNPs were identified. These cSNPs resulted in nonsynonymous amino acid changes or gain and loss mutations of start or stop codons in 325 genes (Table S16a). Additionally, 173 nonsynonymous-mutation-related genes were found to be enriched in 25 pathways (*P* < 0.05) (Table S16b), which included oxidative phosphorylation, ether lipid metabolism, DNA replication, repair, and recombination, and many others. Furthermore, these genes were enriched (*P* < 0.05) in 60 GO terms related to fatty acid beta-oxidation, cytoplasm, quinone binding, and many others (Table S16c).

The majority (78.57%, 22/28) of the clades appeared to signal cross-ward transmissions, and only six clades were detected in a single ward. Twenty-one (75.00%) clades, including 514 isolates (89.55% of the ST2 isolates), were considered to be nosocomial outbreaks based on the standard of infecting more than four patients and appearing in more than one ward. Conversely, three clades (21.42%) and the remaining unclustered ST2 isolates, including 41 (7.14%) isolates, were considered as opportunistic clades under the standard of appearing in a few wards (one or two) and infecting less than four patients (Table S5). From this perspective, it can be concluded that the overwhelming majority (416/487, 85.42%) of the *A. baumannii* infections occurred within the hospital, with the exception of the possible initial carriers of each clade.

### Isolates with close genetic distance are spatially and temporally linked

**Fig 6:**
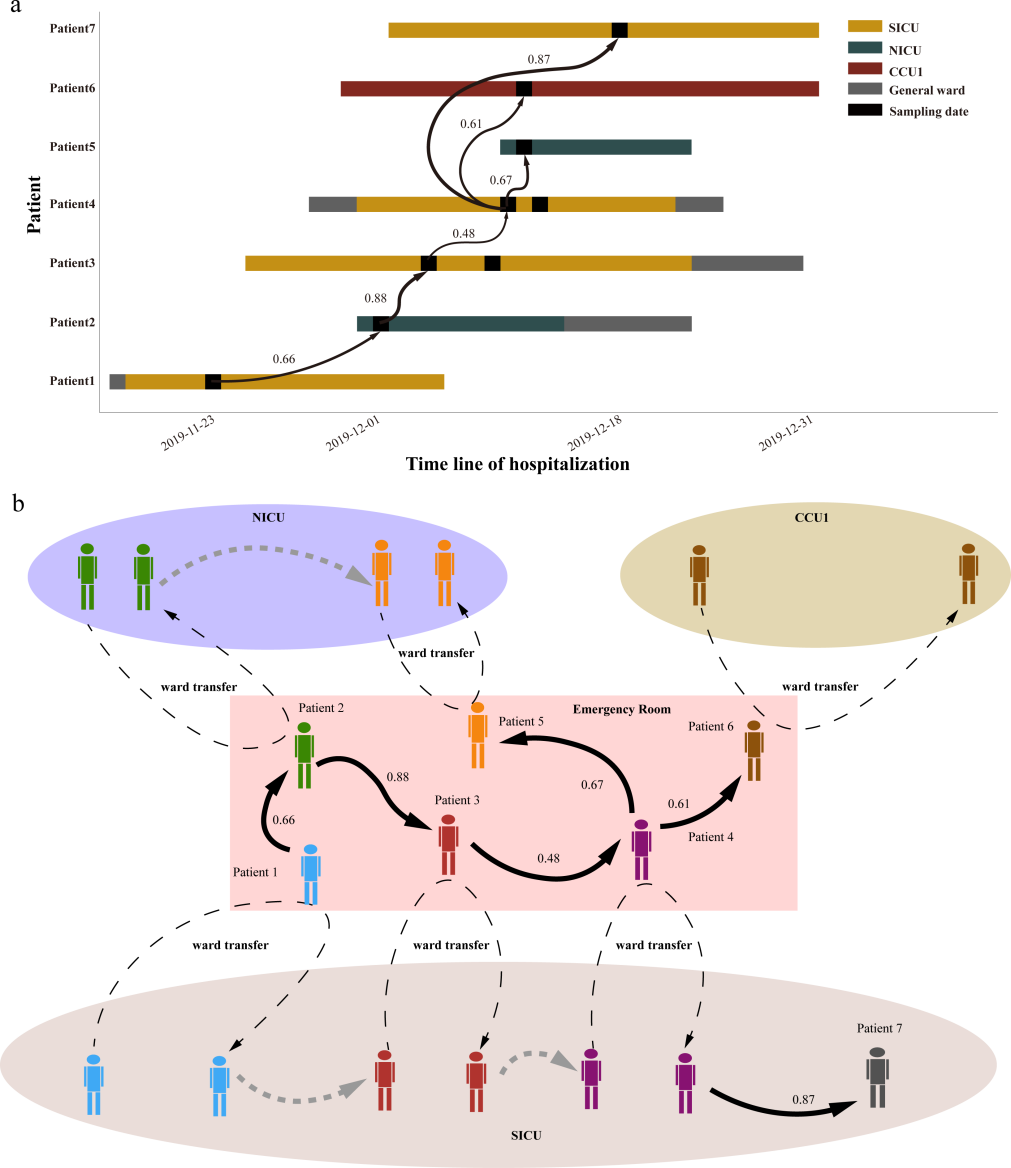
The simulated transmission progress of ST2-4 isolates in hospital. (a) The time and ward span of the patients infected with ST2-4 *A. baumannii*; ST2-4 clade consists of nine isolates from seven patients that were hospitalized in three wards and was defined as the recent group since its isolation time spanned the last 2 months of the sampling period; (b) The simulated transmission progress according to the epidemiological investigation of each patient.

After simulating the transmission of isolates, the entire clades of ST2-4 and ST2-20, as well as the majority of the ST2-6 and ST2-7 clades (Table S17), were reconstructed. ST2-4 emerged during the final 2 months of the sampling period and infected seven patients across three wards within 25 days, which is typical of the early stages of breakout. Four chains, including patient1-patient2, patient2-patient3, patient4-patient5, and patient4-patient6, revealed high possibilities of cross-ward transmissions. Further analysis of their medical histories revealed that these patients were spatially and temporally linked in an emergency ward (Fig. 6). This suggests that the transfer of patients across wards was a significant factor in contributing to the cross-ward transmission of *A. baumannii*. Therefore, implementing higher levels of disinfection and stricter disinfection strategies in the cross-regions of different wards is crucial.

The transmissions of the ST2-20 clade appear to be specific to the ward, as this clade only contained eight isolates from five patients who were all hospitalized in the surgical intensive care unit within a period of 1.5 months (Fig. S13a). In contrast to the short epidemic time span of the ST2-4 and ST2-20 clades, the ST2-6 and ST2-7 clades had much longer epidemic timescales, spanning approximately 9 and 11 months, respectively. Despite incomplete transmission routes in the ST2-6 and ST2-7 clades, inter-ward transmission was still observed in both clades (Fig. S13c and d).

### Genomes evolved under long-term hospital environment selection

After a thorough examination of the epochs of occurrence for each clade, significant differences were identified between these ST2 clades. Comparison of LPG and SPG groups revealed strong genetic consistency of different genotypes at 1,076 allelic loci. Furthermore, the genotypes of 836 loci in the LPG group were also dominant in the RG group (Table S18). These loci were linked to 97 genes, and 44 of them had nonsynonymous mutations, which included one ARG and seven VFGs (Table S19). Five VFGs were functionally classified as immune evasion in the VFDB, suggesting that adaptive evolution in immune evasion mechanisms might contribute to pathogen survival in the host and increase the risk of patient-to-patient transmission. However, the risk of immunological invasion caused by epitope variation region was minimal as no difference in ST2 epitope region was observed (Table S20a and b).

In addition to SNP-level variations, *F3P16_RS06935*, *F3P16_RS06940*, and *F3P16_RS12730* were found to be absent at high frequencies (>65%) in the SPG group but present at high frequencies (>95%) in the LPG and RG groups (Table S19). *F3P16_RS06935* encodes a major facilitator superfamily transporter, which is an efflux pump that is associated with multi-antibiotic resistance in *A. baumannii* (36, 37). *F3P16_RS06940* encodes a TetR/AcrR family transcriptional regulator that regulates the expression of the efflux pump, and *F3P16_RS12730* is a cyclohexanone monooxygenase that catalyzes an oxygen insertion reaction on cyclohexanone to form a seven-membered cyclic product, epsilon-caprolactone (38).

**Fig 7:**
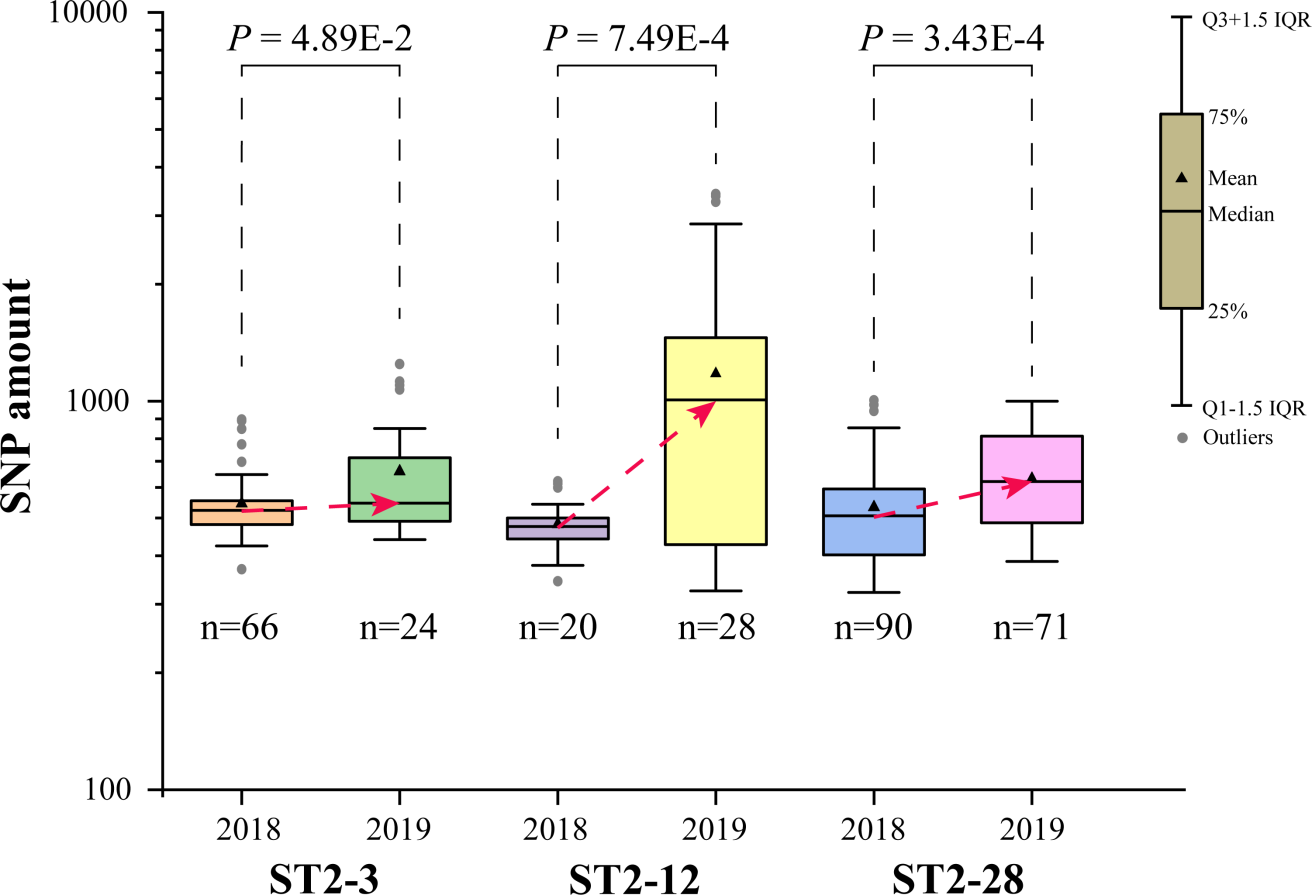
Boxplot showing mutation accumulation of three ST2 clones. For each clone, the first isolate was set as the reference, the following isolates were compared to the reference genome to identify the SNP divergence. The isolates were grouped by the isolation time for comparison. Student’s *t*-test was used to compare the difference of different groups.

To investigate whether mutations increased over time, we conducted a comparison of SNP divergence between the initial isolate and subsequent isolates collected over time. We focused our analysis on three predominant ST2 clones with the highest number of strains: ST2-3 (*n* = 90), ST2-12 (*n* = 48), and ST2-28 (*n* = 161). For each of these clones, we selected the first isolate as the reference and calculated the SNP count between this reference and every subsequent strain in the respective clone. We then categorized these strains into two distinct groups based on their year of isolation, specifically 2018 and 2019. Remarkably, all three ST2 clones from the 2019 group exhibited a significantly higher number of SNPs when compared to their counterparts from the 2018 group. This finding highlights a substantial accumulation of mutations over time, as depicted in Fig. 7.

To establish a more comprehensive understanding of the connections between antibiotic phenotypes and genetic mutations, we conducted a genome-wide association study (GWAS) analysis to explore the relationship between specific SNP variations and antibiotic resistance. We discovered 176 loci demonstrating significant correlations with nine distinct antibiotics (Table S21). These SNPs were linked to 35 genes, comprising 19 missense mutations, 43 synonymous mutations, and 10 SNPs located upstream of corresponding genes. The 19 missense mutations were associated with 14 genes and were found to be correlated with the resistance of eight antibiotics (Table 2). Notably, none of these identified genes were previously recognized as ARGs.

**TABLE 2:** GWAS revealed missense SNPs associated with antibiotic resistance

Gene locus	Gene symbol	SNP alt	Amino acid change	Associated antibiotics^*a*^	Gene description
F3P16_RS00065	−	c.62C > T	p.Ala21Val	CRO	Anhydro-N-acetylmuramic acid kinase
F3P16_RS01705	*yccS*	c.304G > C	p.Ala102Pro	CRO	YccS family putative transporter
F3P16_RS01830	*glyS*	c.379G > A	p.Asp127Asn	CTT, CXM, NIT	Glycine—tRNA ligase, beta subunit
F3P16_RS01995	−	c.700A > T	p.Ile234Phe	CRO	Metal-dependent hydrolase
F3P16_RS05245	*fabR*	c.668G > C	p.Arg223Pro	FEP	HTH-type transcriptional repressor FabR
F3P16_RS06490	−	c.1225G > A	p.Val409Met	CRO	M48 family metalloprotease
F3P16_RS07455	−	c.593C > T	p.Ala198Val	CTT, CXM, NIT	Hypothetical protein
F3P16_RS08530	−	c.666T > A	p.Phe222Leu	CRO	Acyl-CoA dehydrogenase family protein
F3P16_RS10050	−	c.608C > T	p.Ala203Val	CRO	Adenylate/guanylate cyclase domain-containing protein
F3P16_RS10215	*rpsA*	c.1561G > A	p.Ala521Thr	CRO	30S ribosomal protein S1
F3P16_RS11440	−	c.344A > C	p.Asp115Ala	CRO	UTRA domain-containing protein
F3P16_RS13330	−	c.4041T > G	p.Asp1347Glu	CIP, CTT, CXM, IMP, NIT	Tape measure protein
		c.4017A > T	p.Leu1339Phe	CIP, CTT, CXM, IMP, NIT	
		c.3940G > A	p.Asp1314Asn	CIP	
F3P16_RS13415	−	c.220C > G	p.Gln74Glu	CTT, CXM, NIT	Hypothetical protein
		c.211G > A	p.Asp71Asn	CTT, CXM, NIT	
		c.202T > G	p.Ser68Ala	CTT, CXM, NIT	
		c.181A > G	p.Ile61Val	CTT, CXM, NIT	
F3P16_RS18380	−	c.1684G > A	p.Asp562Asn	GEN	Polysaccharide biosynthesis tyrosine autokinase

^*a*^ CIP, ciprofloxacin; CRO, ceftriaxone; CTT, cefotetan; CXM, cefuroxime; GEN, gentamicin; IMP, imipenem; NIT, nitrofurantoin.

## DISCUSSION

Based on the MLST typing results of both public and private data, we observed a divergent epidemic prevalence of *A. baumannii* across various countries and continents. Notably, China had a significantly higher prevalence of ST2 than other countries or regions, with ST2 being highly dominant in ICUs, which is consistent with previous studies (12, 39). This finding can be attributed to the fact that the majority of the ST2 are MDR and China has been reported to have one of the highest levels of antibiotic overuse (40). In our study, ST2 strains exhibited an extremely high rate of resistance to aminoglycosides, carbapenems, and cephems, which is consistent with the high presence of corresponding ARGs in their genomes. Surprisingly, only three antibiotics, trimethoprim-sulfamethoxazole (60.08%), cefoperazone-sulbactam (63.90%), and tigecycline (49.5%), showed a lower rate of resistance in the AST trials. This is concerning because these antibiotics are often used as priority drugs in current MDRAB therapy, and once these barriers are breached, few effective treatment options remain.

Obviously, the explosion of ST2 greatly contributed to the global prevalence of *A. baumannii*. Tracking the phylogenetic relationship of global and Chinese hospital isolates, the hospital endemic *A. baumannii* clades were discovered to have multiple origins. In addition, comparison of the ST2 in 15 Chinese hospitals confirmed that different ST2 clades can outburst in different hospitals and might have evolved to be hospital-specific in different hospitals. Meanwhile, two evident breakouts of same clade in different hospitals were observed, which suggested that hospital-specific *A. baumannii* can cause outbreak in other hospitals. The transmission pathways of these clones could be direct, like patient transfers between hospitals, or they could be indirect, involving secondary transmission through the community. Further research is needed to explore this aspect. Fortunately, these two cross-hospital breakouts of same clade only appeared in the same city, and wider spread was not observed.

In this study, spatially and temporally correlated isolates with close genetic distances were observed within the hospital setting. Both inter-ward and intra-ward transmission chains were identified, and a retrospective analysis of the complete transmission chain of the ST2-4 clade revealed that cross-ward patient transfer plays a crucial role in the nosocomial transmission of pathogens. To break this transmission chain, physical isolation through the implementation of separate wards should be the most effective measure as contaminated hands, medical instruments, and object surfaces are significant contributors to the widespread transmission of *A. baumannii* (41). However, due to limited medical resources, it may be more practical for most hospitals to strictly enforce disinfection procedures and isolation monitoring before patient transfer.

Based on the phylogenetic tree and cSNP divergence, minimal differences were observed within the ST2. However, our study identified hotspots of variation in 373 coding genes that were enriched in 20 GO terms and 10 KEGG pathways, which included drug resistance and the metabolism of various amino acids (Table S17 to S19; Fig. S8 and S9). These genetic variations likely resulted from adaptive evolution to the complex hospital and intra-patient environments. Nonetheless, it is important to note that the prevalence of hospital-specific clades in a particular hospital does not necessarily imply that they will not be present in other hospitals. Highly evolved clades have also been observed to be prevalent in different hospitals, indicating the existence of cross-hospital transmission chains.

Compared to previous studies, our research utilized a higher resolution of genotyping through whole genomic variation comparison. This enabled us to investigate the transmission and evolution of *A. baumannii* at a sub-ST level. In our study, we identified 28 clades based on cSNP discrepancies, with the majority (95.82%) of ST2 falling into these clades. Only 32.14% of these clades were classified as prolonged-term existing clones, which infected 61.6% (300/487) of the patients. This finding confirmed that the primary hospitalized infectious clades were relatively conservative. At the same time, we observed that these conservative clades were also evolving, as mutations were observed increasing over time. And we believe a portion of mutations should result from the environmental selection pressure. To further explore the evolution of ST2 clades, we categorized them into three groups based on the time of their appearance. We hypothesized that the adapted clades would have undergone genetic changes that allowed them to thrive in the hospital or intra-host environments, while the non-adapted clades remained unchanged. Specifically, the LPG isolates should have evolved to be more adaptable, whereas the SPG isolates should not have. We compared the LPG and SPG groups to identify loci that underwent substantial adaptation and validated these findings in the RG group. Our analysis revealed significant genotypic differences at 836 loci, including nonsynonymous mutations in 44 genes, of which seven were VFGs. Additionally, we observed copy number variations in genes associated with antibiotic resistance between different groups. Notably, 71.43% of the involved VFGs were classified as immune evasion in VFDB, indicating that immune evasion might result in stronger transmissibility. These results provided evidence that long-term existing ST2 clones had evolved to adapt to the hospital environment, while emerging opportunistic clades might fail to survive from constant disinfection and antibiotic use. Therefore, to better control nosocomial infections, changing or combining disinfectant use may be a potentially effective approach.

SNP variations associated with antibiotic resistance phenotypes have been documented in a range of pathogens (42, 43). Notably, *A. baumannii* has also shown instances of SNP-related resistance variants. A case in point involves amino acid substitutions within *gyrA* and *parC*, which can significantly affect the quinolone resistance (44). In our study, we conducted a GWAS analysis to explore the potential links between these SNP variations and antibiotic phenotypes. Due to the formidable resistance exhibited in multiple antibiotics, our analysis identified only 19 missense SNP loci with strong statistical significance. These variations may indeed impact *A. baumannii*’s antibiotic resistance, but further experimental validation is imperative.

In conclusion, we conducted a 2-year surveillance investigation of *A. baumannii* isolates from seven different ICUs in a large Chinese general hospital. In this study, we comprehensively investigate the genomic evolution and transmission of *A. baumannii* in inter- and intra-intensive care units with both a long-time scale and a large sample size. Our findings will contribute to understanding the molecular evolution mechanism and transmission mode of this pathogen, and provide valuable insights for further nosocomial infection control and antibiotic therapy. However, since our study was based on retrospective samples, corresponding nosocomial environmental samples were not available, resulting in the inability to trace transmission chains of different ST2 subtypes and uncover transmission chains between patients, healthcare workers and the hospital environments. More comprehensive samples are needed to promote further detailed investigation on nosocomial transmission monitoring.

## Data Availability

The sample information, raw data and assembled genome sequences were uploaded to National Genomics Data Center (NGDC) (https://ngdc.cncb.ac.cn/) with the project number PRJCA008869. The sequenced NGS reads were submitted to the GSA database of NGDC under accession number CRA006514. The other data source used in this study is described in supplemental Table S4.

## SUPPLEMENTAL MATERIAL

Supplemental figures spectrum.02207-23-s0001.docx Fig. S1 to Fig. S13.

Supplemental tables spectrum.02207-23-s0002.xlsx Tables S1 to S21.

ASM does not own the copyrights to Supplemental Material that may be linked to, or accessed through, an article. The authors have granted ASM a non-exclusive, world-wide license to publish the Supplemental Material files. Please contact the corresponding author directly for reuse.
