# Supplementary material for: Nosocomial surveillance of multidrug-resistant Acinetobacter baumannii: a genomic epidemiological study
Source: Microbiol Spectr. 2024 Jan 10;12(2):e02207-23. doi: 10.1128/spectrum.02207-23 (PMC10846281; doi:10.1128/spectrum.02207-23)
Supplement: Supplemental figures — Fig. S1 to Fig. S13. [file spectrum.02207-23-s0001.docx]

Supplementary Figures


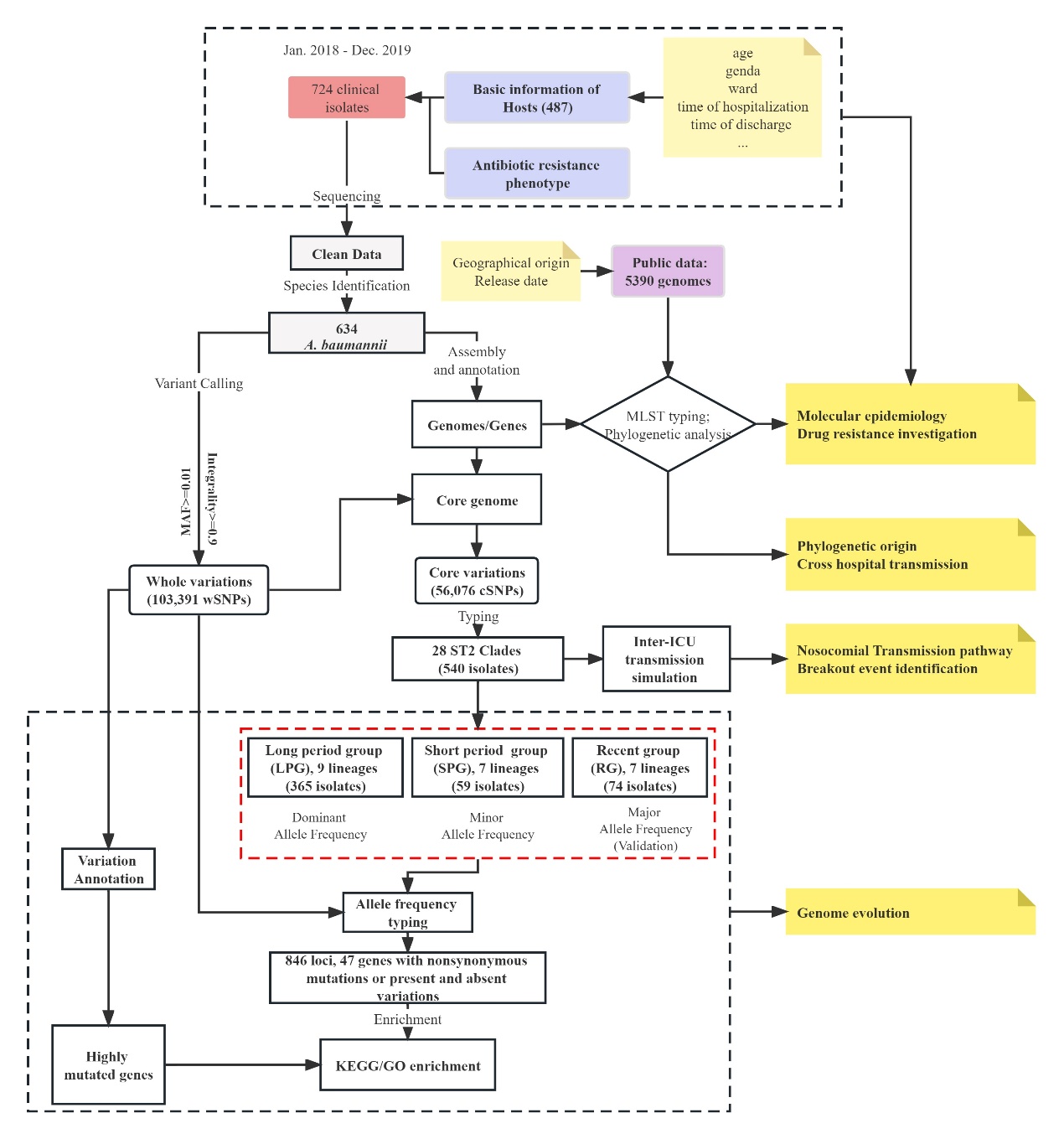


Figure S1 Flow chart of the study design and analysis


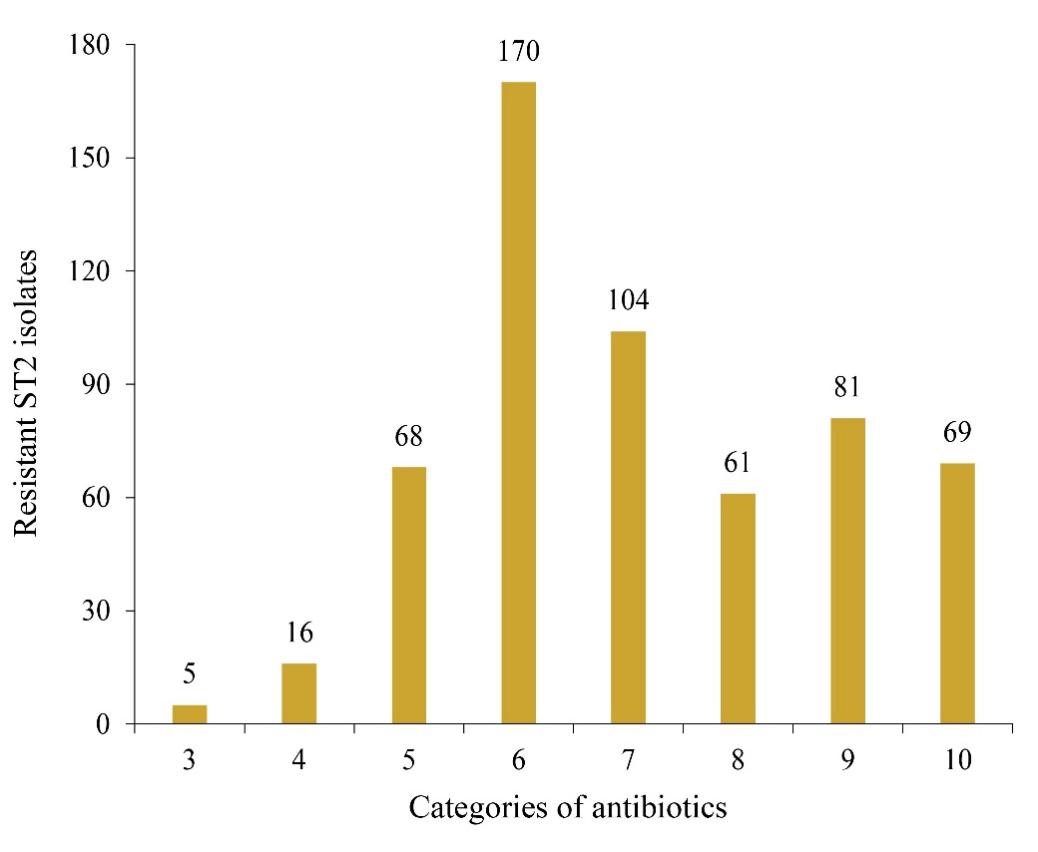


Figure S2 Histogram showing the antibiotic resistance of ST2 isolates

A total of nineteen antibiotics corresponding to ten categories were used to conduct the antibiotic susceptibility test. This figure shows the distributions of the resistant categories of ST2 isolates.


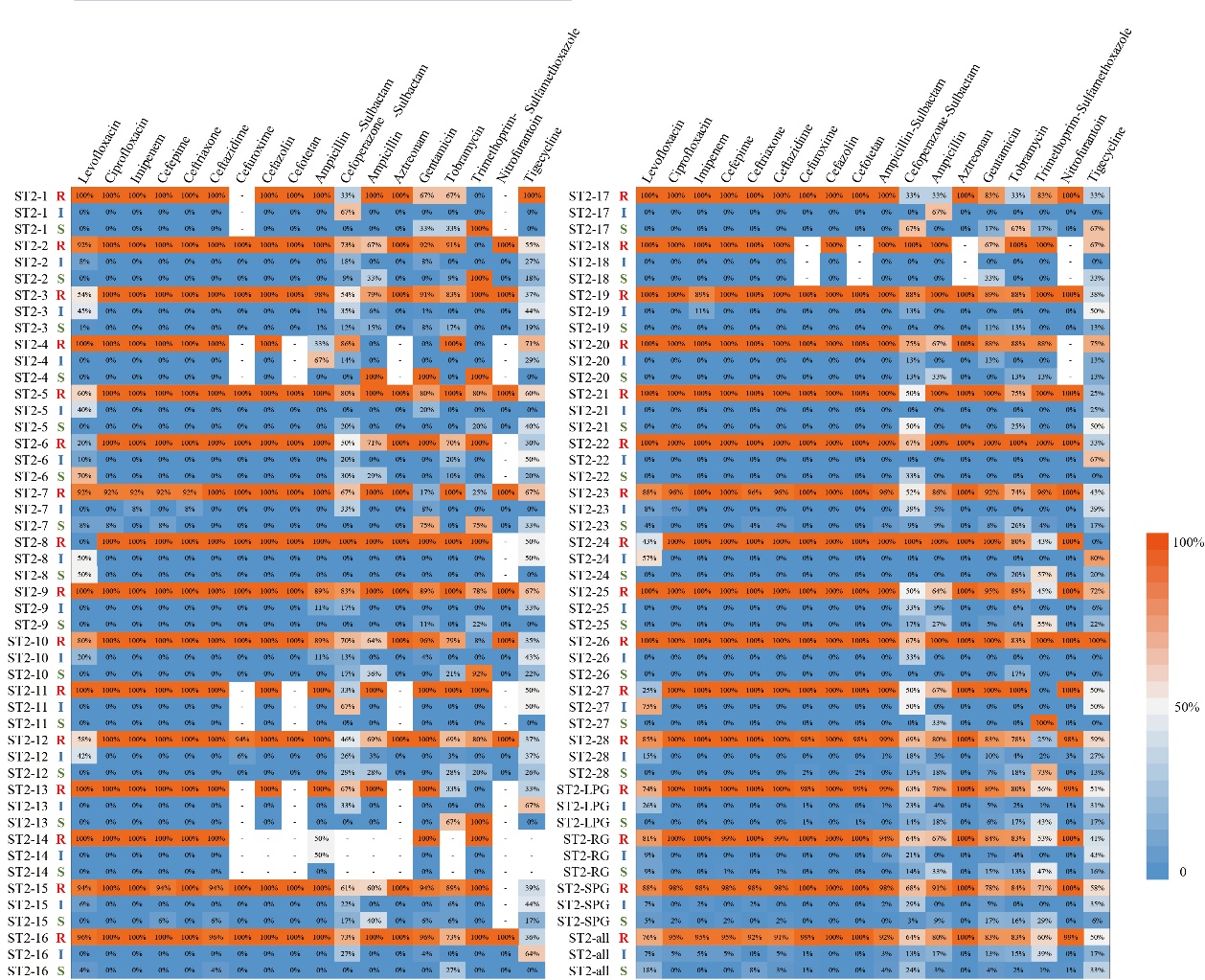


Figure S3 Antibiotic resistance of ST2 isolates

“R”, “S”, “I” means “resistant”, “sensitive” and “intermediate” to the corresponding antibiotics; the white blank of the ASTs region means the phenotype data were missing.


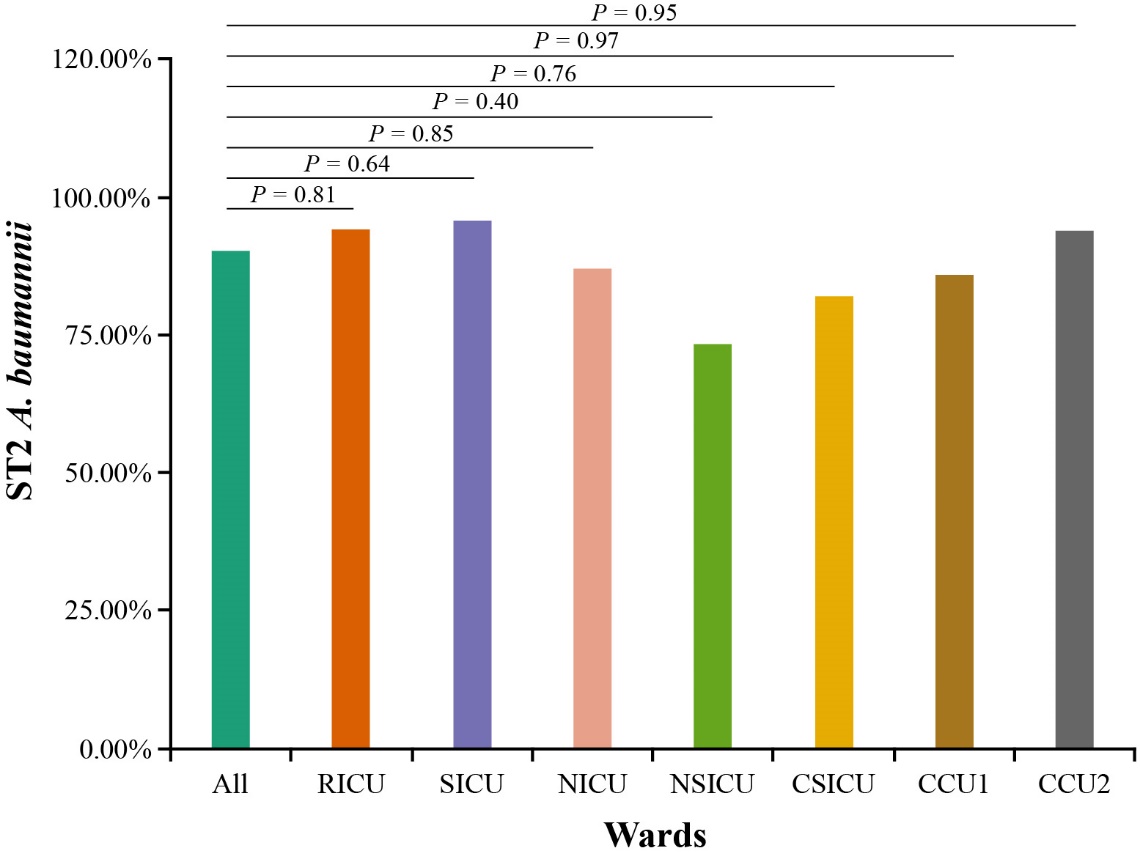


**Figure S4 Distribution of ST2** **isolates in each ward**

The histogram shows the percentage of ST2 in each ward and chi-squared test was used to analysis the divergence between each ward and the whole dataset.


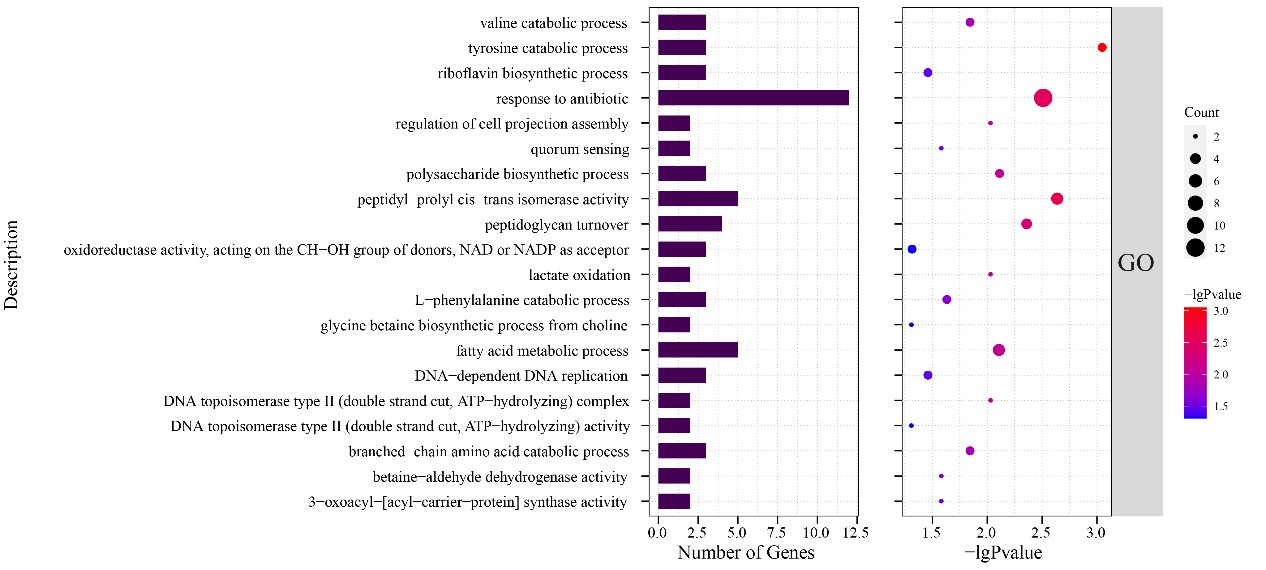


Figure S5 GO enrichment of highly mutated genes of ST2 isolates

A total of 373 severely altered genes were identified within ST2, these genes were enriched in 20 Gene Ontologies (GO), including GO:0046677 that response to antibiotics (*P =* 0.0031).


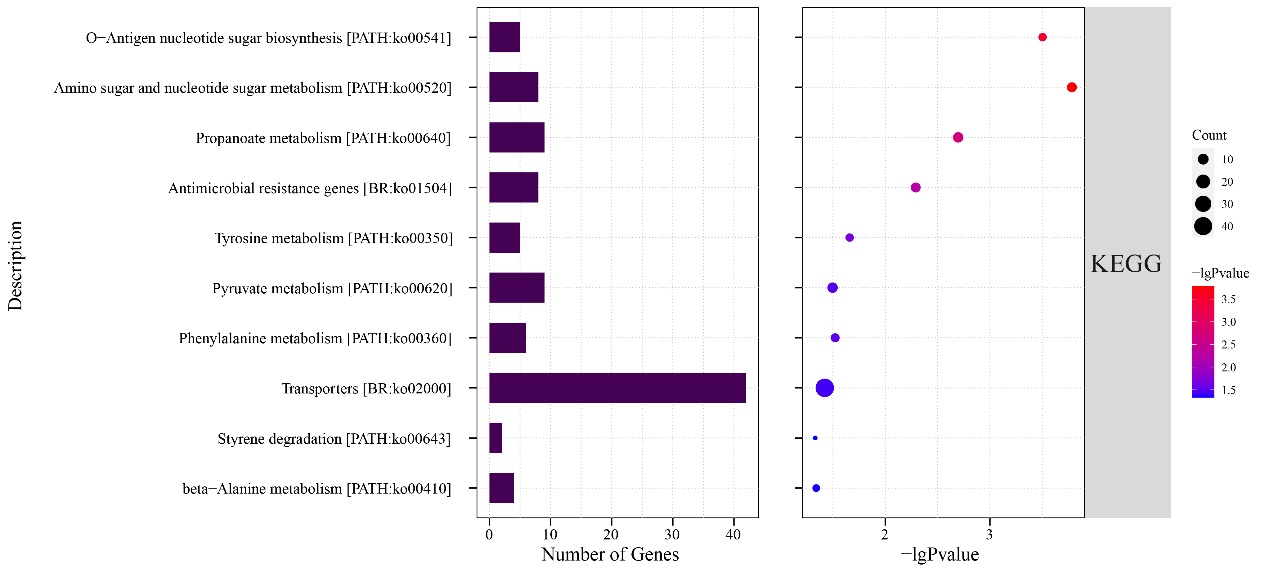


Figure S6 KEGG enrichment of highly mutated genes of ST2 isolates

A total of 373 severely altered genes were identified within ST2 isolates, these genes were enriched in enriched in 10 KEGG pathways, including KO01504 (*P* = 0.0051) that related to antimicrobial resistance.


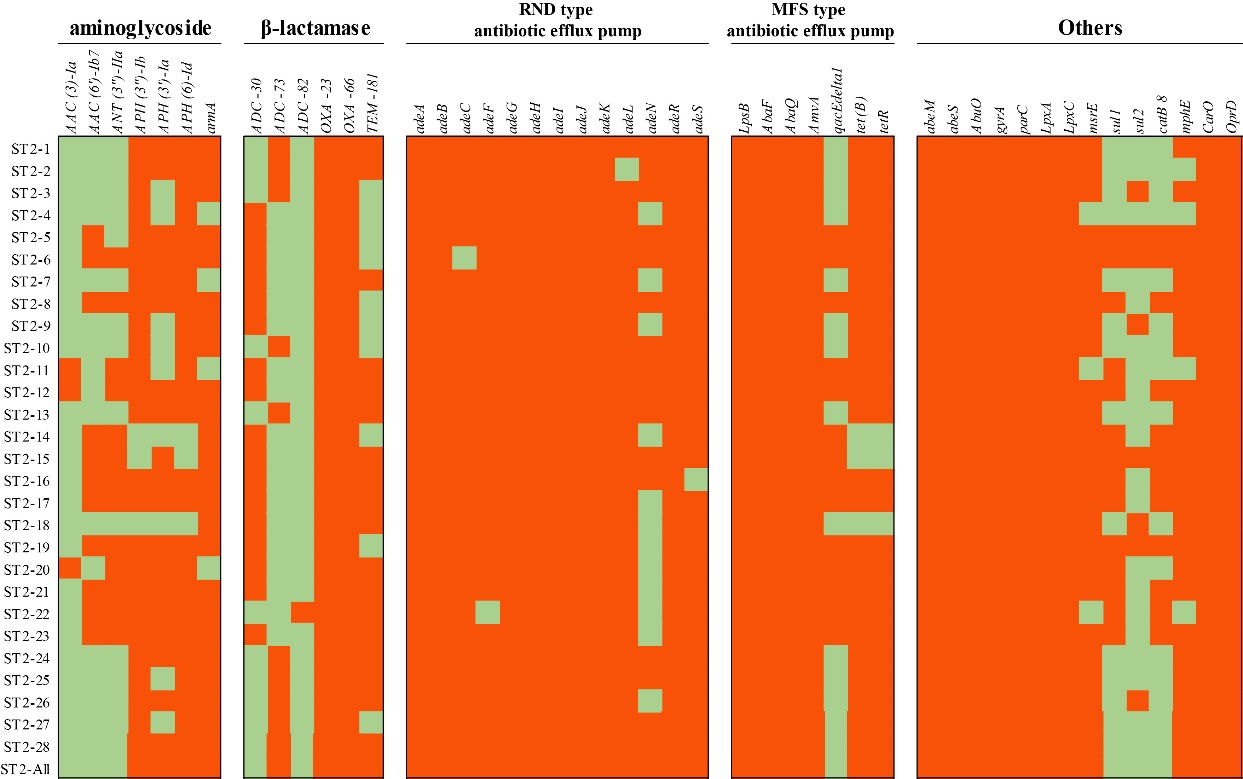


Figure S7 Heatmap showing the ARGs profiles of ST2 isolates.

The gene names and categories were printed in the top of the heatmap; the *y* axis shows the group names; the heatmap shows the existence of the ARGs in each group, with orange color showing the presence and light green color represents the absence.

**
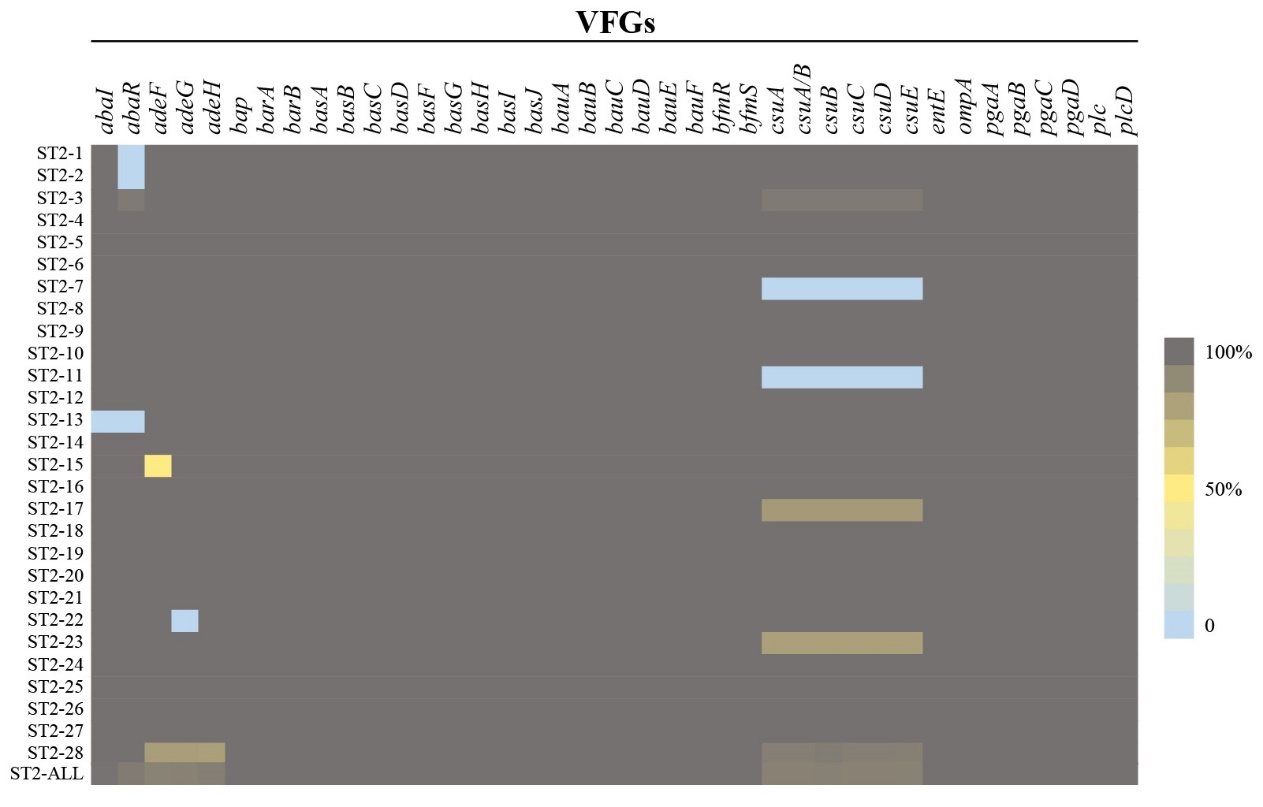
**

Figure S8 The absence and presence of VFGs in ST2 isolates

The gene names were printed in the top of the heatmap; the y axis shous the group names; the heatmap shows the existence of the VFGs in each group, with a color bar on the right.

**
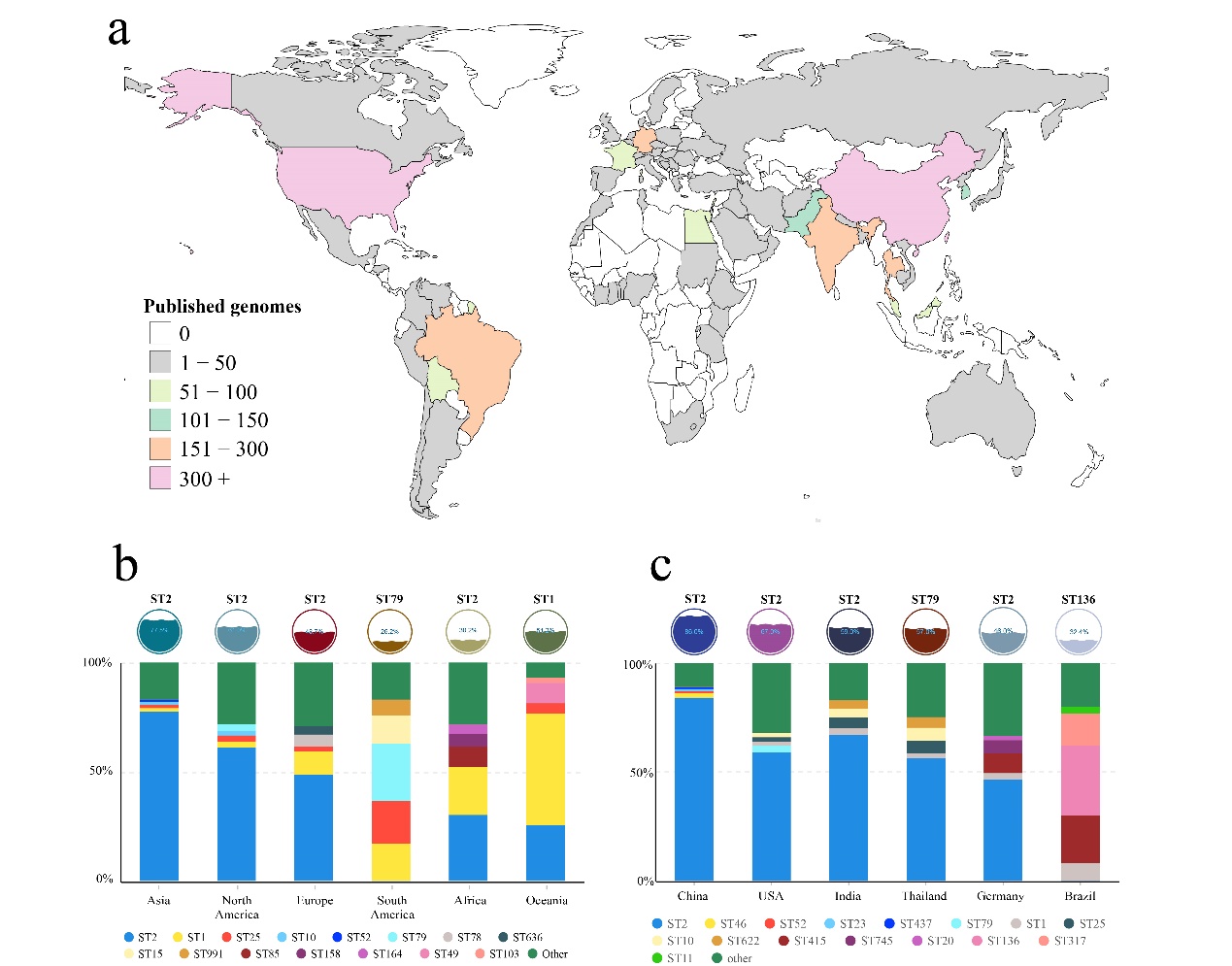
**

**Figure S9 Epidemiology of worldwide *A. baumannii***

a. The geographical distribution of the published *A. baumannii* genomes used for this study; b. The statistics of the MLST typing result of the worldwide isolates. ST2 was the most popular sequence types in Asia (77.5%), North America (61.0%), Europe (48.7%) and Africa (30.2%), while ST79 and ST1 were most popular sequence types in South America (26.2%) and Oceania (51.2%), respectively; c. The statistics of the MLST typing result of the top six countries.


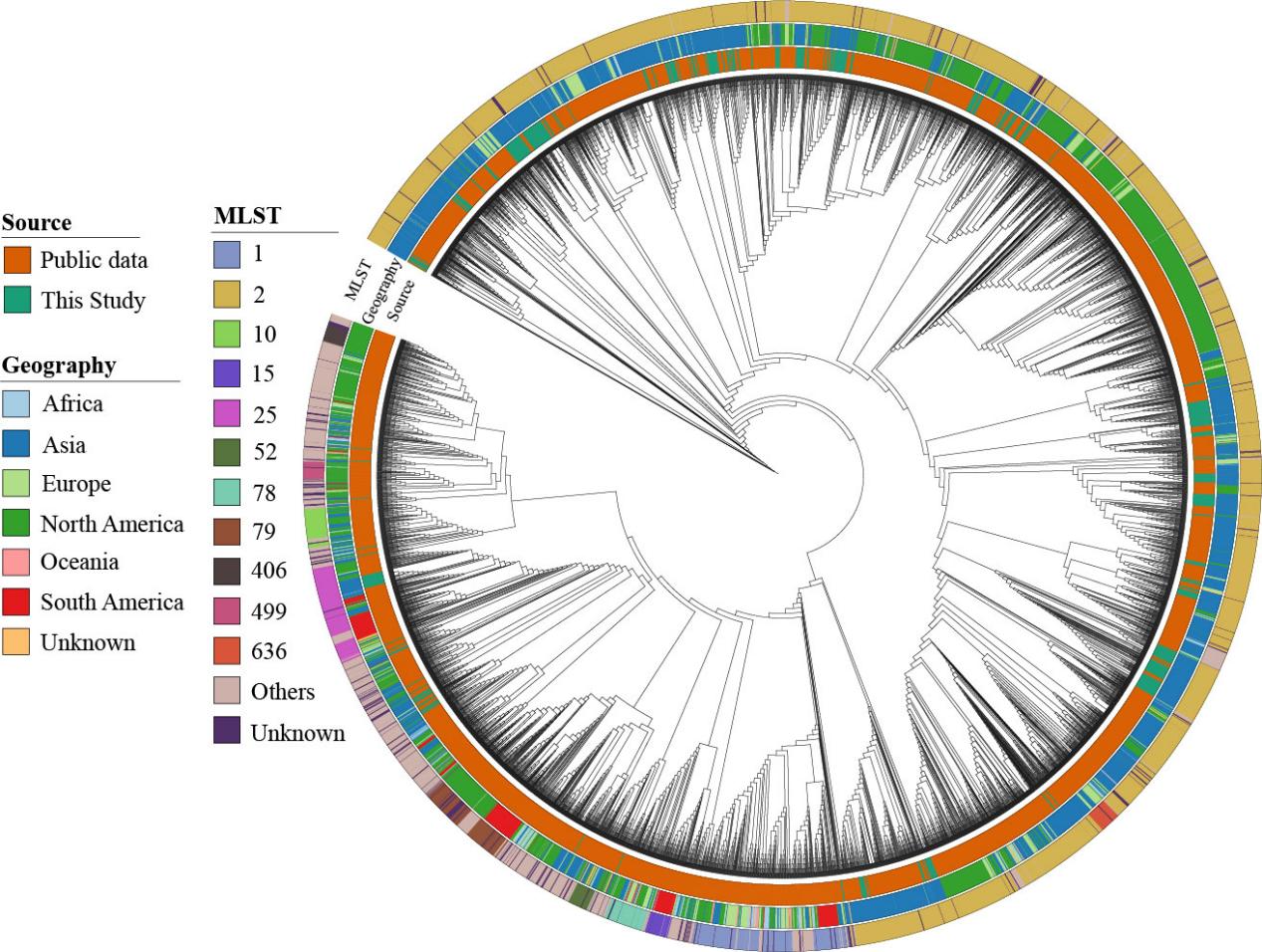


Figure S10 Phylogenetic tree showing the 6,024 worldwide isolated *A. baumannii*

The ML tree was constructed using protein sequence of the core genes of 6,024 *A. baumannii*. The outer circle shows the MLST types, the geography and the data source of the strains from outside to inside.


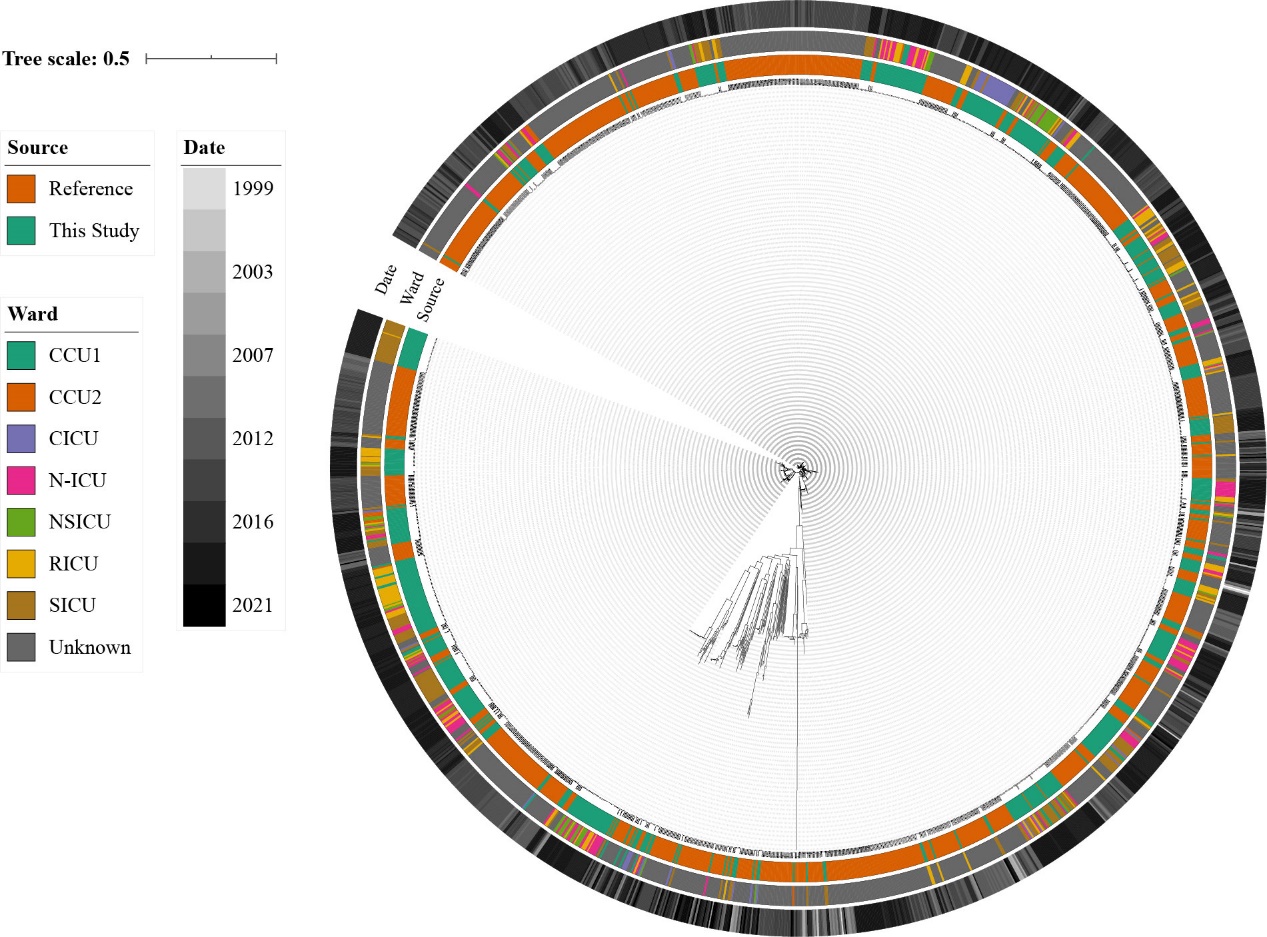


Figure S11 Phylogenetic tree of *A. baumannii* from China

The ML tree was constructed using protein sequence of the core genes of 6,024 *A. baumannii,* showing all the *A. baumannii* from China. The outer circle shows the data release date, ward source and public or private data source of the strains from outside to inside.


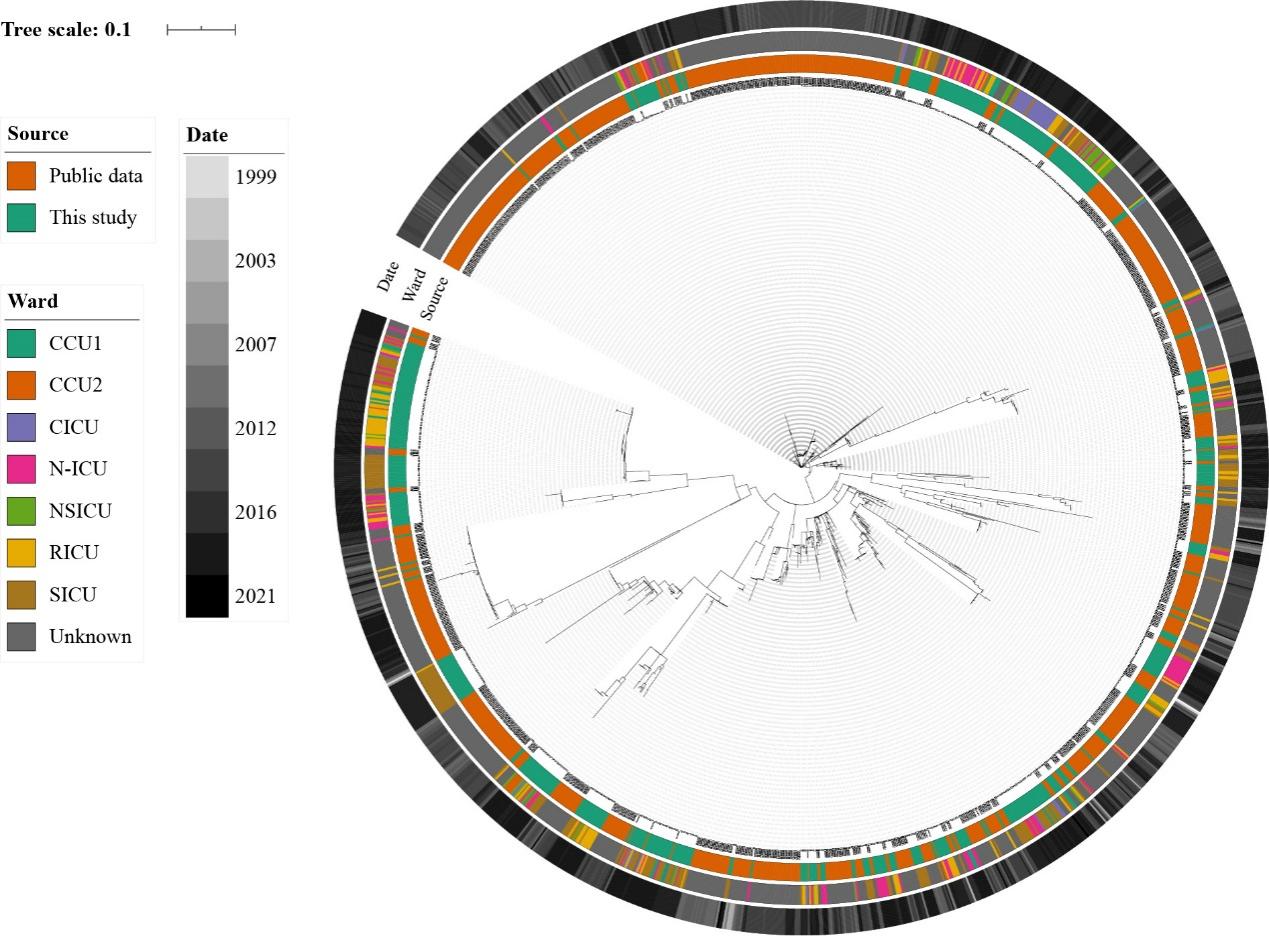


Figure S12 Phylogenetic tree of ST2 *A. baumannii* from China

The ML tree was constructed using protein sequence of the core genes of 6,024 *A. baumannii,* showing all the ST2 *A. baumannii* from China. The outer circle shows the data release date, ward source and public or private data source of the strains from outside to inside.


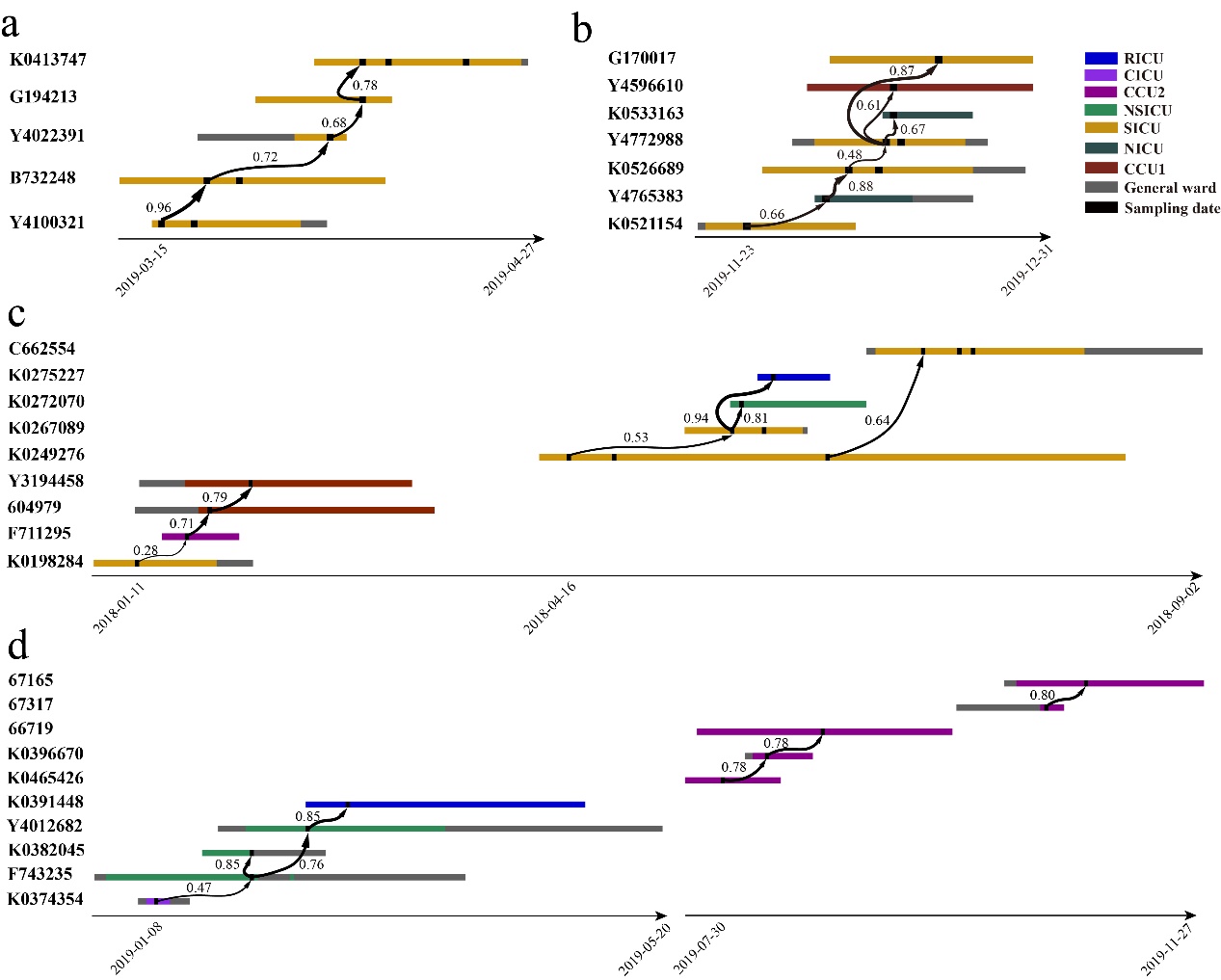


Figure S13 Simulated transmission route of ST2-4, ST2-6, ST2-7 and ST2-20 clades

a. Simulated model of intra-ward transmission with ST2-20 *A. baumannii*. ST2-20 consist of eight isolates from five patients. b. the time and ward span of the patients infected with ST2-4 *A. baumannii*. ST2-4 clade consists of nine isolates from seven patients that were hospitalized in three wards and was defined as the recent group since its isolation time spanned the last two months of the sampling period; c. simulated path of cross-ward transmission ST2-6; d. simulated path of cross-ward transmission ST2-7. The whole isolates of ST2-6 and ST2-7 were contained, but the transmission chains were incomplete probably due to sampling history.
